# CYP3A5 unexpectedly regulates glucose metabolism through the AKT–TXNIP–GLUT1 axis in pancreatic cancer

**DOI:** 10.1016/j.gendis.2023.101079

**Published:** 2023-09-07

**Authors:** Ming Shao, Qingfei Pan, Haiyan Tan, Jing Wu, Ha Won Lee, Andrew D. Huber, William C. Wright, Ji-Hoon Cho, Jiyang Yu, Junmin Peng, Taosheng Chen

**Affiliations:** a Department of Chemical Biology and Therapeutics, St. Jude Children's Research Hospital, Memphis, TN 38105, USA; b Department of Computational Biology, St. Jude Children's Research Hospital, Memphis, TN 38105, USA; c Center for Proteomics and Metabolomics, St. Jude Children's Research Hospital, Memphis, TN 38105, USA; d Department of Structural Biology, St. Jude Children's Research Hospital, Memphis, TN 38105, USA; e Department of Developmental Neurobiology, St. Jude Children's Research Hospital, Memphis, TN 38105, USA

**Keywords:** Cell migration, CYP3A5, Glucose metabolism, GLUT1, Pancreatic cancer, TXNIP

## Abstract

CYP3A5 is a cytochrome P450 (CYP) enzyme that metabolizes drugs and contributes to drug resistance in cancer. However, it remains unclear whether CYP3A5 directly influences cancer progression. In this report, we demonstrate that CYP3A5 regulates glucose metabolism in pancreatic ductal adenocarcinoma. Multi-omics analysis showed that CYP3A5 knockdown results in a decrease in various glucose-related metabolites through its effect on glucose transport. A mechanistic study revealed that CYP3A5 enriches the glucose transporter GLUT1 at the plasma membrane by restricting the translation of TXNIP, a negative regulator of GLUT1. Notably, CYP3A5-generated reactive oxygen species were proved to be responsible for attenuating the AKT–4EBP1–TXNIP signaling pathway. CYP3A5 contributes to cell migration by maintaining high glucose uptake in pancreatic cancer. Taken together, our results, for the first time, reveal a role of CYP3A5 in glucose metabolism in pancreatic ductal adenocarcinoma and identify a novel mechanism that is a potential therapeutic target.

## Introduction

The *CYP3A5* gene encodes a member of the cytochrome P450 (CYP) superfamily of enzymes, which are monooxygenases that catalyze many reactions involved in drug metabolism and in the synthesis of cholesterol, steroids, and other lipids.^1^, ^2^, ^3^, ^4^ The 3A subfamily of CYPs (CYP3A) is critical for xenobiotic clearance in humans and is reported to metabolize more than half of all currently prescribed drugs.^5^, ^6^, ^7^ Recently, studies of CYP3A5 have addressed the relation of *CYP3A5* single-nucleotide (or genotype) polymorphism to cancer risk or drug metabolism.^6^^,^^8^ It has been reported that CYP3A5 is overexpressed in pancreatic ductal adenocarcinoma (PDAC) and mediates chemoresistance in different subtypes of this cancer.^9^ Importantly, CYP3A5 down-regulation re-sensitizes drug-resistant PDAC cells, confirming the role of elevated CYP3A5 levels in drug resistance. However, this does not explain why CYP3A5 is highly expressed in some tumors even before drug treatment, and the role of CYP3A5 in cancer progression remains largely unknown.

PDAC is a highly aggressive malignancy, the lethality of which stems from a lack of early diagnosis and the limited response of the cancer to treatment.^10^, ^11^, ^12^ It is the most prevalent type of pancreatic neoplasm, developing in the exocrine compartment, and accounts for more than 90% of pancreatic cancer cases.^13^ Despite progress in elucidating PDAC tumor biology and the development of novel therapeutic regimes,^14^^,^^15^ the average 5-year survival rate for PDAC is less than 10%.^16^^,^^17^ A defining characteristic of PDAC is a rewired cellular metabolism that facilitates growth in austere conditions.^15^^,^^18^ The reliance of PDAC on altered metabolic pathways has spawned interest in discovering metabolic vulnerabilities and targeting them for therapeutic gain.^19^^,^^20^

Altered glycolysis has been recognized as the major metabolic hallmark of pancreatic cancer.^21^^,^^22^ Glycolysis is a central carbon metabolism pathway in cells that provides energy in the form of ATP and fuels cell growth and division by providing biomass.^23^^,^^24^ Importantly, the glycolysis branch pathways include several key metabolic pathways, including the pentose phosphate pathway, the hexosamine biosynthesis pathway,^25^ the serine biosynthesis pathway,^26^ and the tricarboxylic acid cycle. Glucose transport across the plasma membrane is one of the rate-limiting steps. Among the glucose transporters (GLUTs), the expression of GLUT1 (encoded by the *SLC2A1* gene) correlates with the progression of PDAC.^19^ GLUT1 expression has been observed to increase gradually as dysplasia increased in severity from low grade to high grade, being detected in 74% of the cases studied, whereas there is no GLUT1 expression in normal pancreatic acini or ducts.^27^ Characterizing the compensations between metabolic pathways and the metabolic dependence between different tumor components helps discover new metabolic drug targets.

In the current study, we focused on the previously unknown physiologic functions of CYP3A5 in tumor progression rather than on its drug metabolism-related effects. We revealed a new role of CYP3A5 in glucose metabolism in PDAC. CYP3A5 was highly expressed in PDAC cells and helped to maintain a high concentration of reactive oxygen species (ROS), which inhibited AKT activation and the phosphorylation of its downstream target 4EBP1. A reduction in 4EBP1 phosphorylation contributes to the decreased translation of TXNIP, a negative regulator of GLUT1 membrane translocation. The increase in GLUT1 localized to the plasma membrane resulted in a higher glucose uptake, which was beneficial for the PDAC cells.

## Materials and methods

### Cell culture

The AsPC-1 cell line (CRL-1682) was purchased from the American Type Culture Collection (ATCC) and grown in RPMI1640 medium (Gibco, 11875-093) supplemented with 10% fetal bovine serum (HyClone, SH30071.03), 1% GlutaMAX (Gibco, 35050-061), and 1% penicillin–streptomycin (Gibco, 15140-122), or in RPMI1640 medium without glucose, glycine, and serine (Teknova, R9660) and supplemented with 10% dialyzed fetal bovine serum (HyClone, SH30079.03IR25), 1% glucose (Gibco, A2494001), and 1% penicillin–streptomycin (Gibco, 15140-122). HPAF-II (CRL-1997) was obtained from ATCC and grown in culture according to the ATCC guidelines.

AsPC-1 CYP3A5-deletion cells (CYP3A5 KO-1 and CYP3A5 KO-2), and the AsPC-1 wild-type (WT) cell line and the CYP3A5-knockout (KO) cell line with inducible CYP3A5 overexpression have been described in our previous publication.^28^

To create AsPC-1 cells with CYP3A5-C441A overexpression, CYP3A5-C441A was introduced by using a QuikChange II XL Site-Directed Mutagenesis Kit (Agilent Technologies, 200521). The PCR-amplified CYP3A5-C441A was cloned into pLVX-TRE3G-ZsGreen at the MIul/EcoRI sites. The 3A5Del-AsPC-1 cells in which CYP3A5-C441A was stably expressed were tested with the P450-Glo CYP3A4 assay (Promega, V9002), in accordance with the manufacturer's protocol. The AsPC-1 CYP3A5-overexpression (CYP3A5-OE) cell line was grown in RPMI1640 medium (Gibco, 11875-093) supplemented with 10% tetracycline-screened fetal bovine serum (HyClone, SH30071.03T), 1% GlutaMAX (Gibco, 35050-061), 2 μg/mL puromycin, and 1% G418.

Cell lines were maintained in humidified CO_2_ incubators at 37 °C, 5% CO_2_. Cells were routinely passaged every 3 or 4 days. Cells were trypsinized using TrypLE Express (Gibco, 12604-013) at 37 °C for 10 min, collected by centrifugation and resuspended in pre-warmed medium, and grown in 75-cm^2^ flasks (Corning, 430641U). The cell density was counted using a Countess cell-counting chamber slide (Invitrogen) and read by a Countess 2 system (Life Technologies). Cell growth was monitored using an Incucyte ZOOM system (Essen Bioscience) that enabled cell images to be acquired inside the incubator, and the cell confluence was calculated by the ZOOM software. All cell lines have been authenticated by short tandem repeat DNA profiling and tested negative for mycoplasma contamination by using the MycoProbe Mycoplasma Detection Kit (R&D Systems).

### RNA interference (siRNA treatment)

Cells were seeded in 6-well tissue culture plates (Corning, 353046). The next day, they were reverse-transfected using Lipofectamine RNAiMAX (Invitrogen, 13778-500). Briefly, 9 μL of RNAiMAX reagent was diluted with 150 μL of Opti-MEM medium (Gibco 31985070), and 3 μL of siRNA (10 μM) was mixed with 150 μL of Opti-MEM medium. Then, 150 μL of the diluted RNAiMAX reagent and 150 μL of the diluted siRNA were mixed and incubated at room temperature (RT) for 5 min, after which 250 μL of the siRNA–lipid complex was added to each well containing cells, and the cells were incubated for 3 days. The siRNAs used (all from Dharmacon) included ON-TARGETplus Human CYP3A5 siRNA SMARTPool (L-009684-01-0005), three different siCYP3A5 siRNAs (J-009684-09-0005, J-009684-10-0005, and J-009684-11-0005), siGENOME Non-Targeting siRNA #5 (D-001210-05) (siNT), siTXNIP (L-010814-00-0005), siGLUT1 (L-007509-02-0005), three different siGLUT1 siRNAs (J-007509-06-0005, J-007509-07-0005, and J-007509-08-0005), and si4EBP1 (L-003005-00-0005). siCYP3A5-5UTR (si5-UTR) was an siRNA specifically targeting the 5′-UTR region of CYP3A5 mRNA, and siCYP3A5-3UTR (si3-UTR) was an siRNA specifically targeting the 3′-UTR region of CYP3A5 mRNA.

### Western blot analysis

Cells were washed with phosphate-buffered saline (PBS), trypsinized, and collected by centrifugation. Protein was extracted using Pierce RIPA lysis buffer (Thermo Scientific, 89900) supplemented with cOmplete Mini protease inhibitor tablets (Roche, 11836153001). The protein concentration was measured with the Pierce BCA Protein Assay Kit (Thermo Scientific, 23227) according to the manufacturer's recommendations. Samples were loaded on 4%–12% NuPAGE Bis-Tris Gels (Life Technologies), subjected to SDS-PAGE, and then dry-blotted onto nitrocellulose membranes using the iBlot Blotting System (Life Technologies). Membranes were blocked using Odyssey blocking buffer (LI-COR Biosciences, 927-70001) at RT for 1 h and then incubated with primary antibodies at RT for a further hour. After being washed with PBS (0.1% Tween 20), membranes were incubated with secondary antibodies conjugated with a species-specific infrared dye (LI-COR) at RT for 1 h. Membranes were scanned with an Odyssey CLx infrared imager (LI-COR). Primary antibodies included anti-CYP3A5 (Abcam, ab108624), anti-actin (Sigma, A5441), anti-GAPDH (Proteintech, 60004-1-Ig), anti-DHFR (Proteintech, 15194-1-AP), anti-PSAT1 (Proteintech, 10501-1-AP), anti-SHMT2 (Proteintech, 11099-1-AP), anti-GLUT1 (Abcam, ab115730) anti-TXNIP (Abcam, ab188865), anti-4EBP1 (Proteintech, 60246-1-Ig), anti-p-4EBP1 (Cell Signaling, 13443S), anti-AKT (Cell Signaling, 2920S), and anti-p-AKT (Cell Signaling, 4060S). Protein bands were quantitated by a ratio of each protein band relative to the lane's loading control using ImageJ.

### CYP3A5 gene expression in primary cancer types and pancreatic cancer cell lines

The RNA-seq FPKM values of CYP3A5 for both tumor and normal samples for 32 human primary cancer types were extracted from The Cancer Genome Atlas cohort (QIAGEN OncoLand, v2019Q1). The fold change in CYP3A5 expression between the tumor and normal tissue for each cancer type was calculated by getDE.limma.2G function from NetBID (v-2.0.2) and visualized by ggplot2 (v-3.3.4). The CYP3A5 expression profiles of 41 pancreatic cancer cell lines were collected from the Cancer Cell Line Encyclopedia cohort (QIAGEN OncoLand, v2019Q1). The log_2_ FPKM values were used for visualization by ggplot2 (v-3.3.4).

### Metabolome profiling by LC–MS/MS

Metabolites were extracted by following our previously optimized protocol.^29^^,^^30^ Briefly, CYP3A5-knockdown (KD) or control AsPC-1 cells were grown in culture in 6-well plates to approximately 85% confluence and washed with 2 mL of ice-cold 1× PBS. The cells were then harvested in 300 μL of freezing 80% acetonitrile (v/v), transferred to 1.5-mL tubes, and lysed in the presence of glass beads in a Bullet Blender (Next Advance) at 4 °C until the sample was homogenized. The resulting lysate was centrifuged at 21,000 g for 5 min, after which the supernatant was dried in a SpeedVac concentrator. The samples were resuspended in 50 μL of 1% acetonitrile plus 0.1% trifluoroacetic acid and separated by Ultra-Micro Spin C18 columns (Harvard Apparatus) into hydrophilic metabolites (flow-through) and hydrophobic metabolites (using an eluent of 125 μL of 80% acetonitrile plus 0.1% trifluoroacetic acid).

Ten microliters of the hydrophilic metabolites were dried, reconstituted in 3 μL of 66% acetonitrile, and analyzed using a ZIC-HILIC column (150 × 2.1 mm) (EMD Millipore) coupled with a Q Exactive HF Orbitrap mass spectrometer (MS) (Thermo Fisher) in negative mode, and the metabolites were eluted within a 45-min gradient (buffer A: 10 mM ammonium acetate in 90% acetonitrile, pH 8; buffer B: 10 mM ammonium acetate in 100% H_2_O, pH 8). Twenty microliters of the hydrophobic metabolites were dried and resuspended in 3 μL of 5% formic acid. They were then analyzed by acidic-pH reverse-phase liquid chromatography with tandem mass spectrometry (LC–MS/MS) with a self-packed column (75 μm × 15 cm with 1.9-μm C18 resin, from Dr. Maisch GmbH) coupled with a Q Exactive HF Orbitrap MS (Thermo Fisher) in positive mode, and the metabolites were eluted within a 50-min gradient (buffer A: 0.2% formic acid in H_2_O; buffer B: 0.2% formic acid in acetonitrile). MS settings for both types of samples included MS1 scans (120,000 resolution, 100–1000 m/z, 3 × 10^6^ AGC, and 50 ms maximal ion time) and 20 data-dependent MS2 scans (30,000 resolution, 2 × 10^5^ AGC, 45 ms maximal ion time, HCD, stepped NCE (50, 100, 150), and 20 s dynamic exclusion). A quality control sample was injected at the beginning, middle, and end of the samples to monitor the signal stability of the instrument.

The data analysis was performed by a recently developed software suite, JUMPm.^31^^,^^32^ Raw files were converted to mzXML format before peak feature detection was performed for individual samples and feature alignment across samples. Metabolite identifications were supported by matching the retention time, accurate mass/charge ratio, and MS/MS fragmentation data to our in-house authentic compound library or by matching them to a downloaded experimental MS/MS library (MoNA, https://mona.fiehnlab.ucdavis.edu/), an *in silico* database generated from the Human Metabolome Database (HMDB), and mzCloud (https://mzcloud.org) based on an accurate mass/charge ratio and MS/MS spectrum. Peak intensities were used for metabolite quantification. The data were normalized by both the cell numbers (before data collection) and the trimmed median intensity of all features across the samples (after data collection). Metabolites identified by in-house authentic compound library were reported in Table S1.

### RNA extraction, sequencing, and data analysis

The total RNA was extracted from both WT and CYP3A5-KD AsPC-1 cells using a Maxwell 16 LEV simplyRNA Tissue kit with a Maxwell 16 Research instrument (Promega) according to the manufacturer's protocol. The RNA concentration was determined with a NanoDrop 8000 spectrophotometer (Thermo Fisher Scientific).

A TruSeq Stranded mRNA LTSample Prep Kit (Illumina) was used for library preparation, and 100-bp paired-end sequencing was performed using an Illumina HiSeq X Ten instrument (Illumina). All relevant sequencing data are available at GEO (GSE149000). The adapters used in the library preparation were identified by FastQC (v-0.11.5) (https://www.bioinformatics.babraham.ac.uk/projects/fastqc/) and trimmed from the raw reads by cutadapt (v-1.13) (https://doi.org/10.14806/ej.17.1.200), using the default parameters. RSEM (v-1.3.0),^33^ coupled with Bowtie2 (v-2.2.9)^34^ was used to quantify the expression of genes and transcripts based on the reference genome hg38 (GRCh38) with gene annotation from GENCODE (release v28). Raw gene-level counts were normalized to counts per million (CPM) and further transformed into log_2_(CPM + 1). Principal component analysis was performed by NetBID (v-2.0.2)^35^ to assess the overall similarity between samples. Differential expression analysis was conducted using the limma R package (v-3.42.2).^36^ Gene set enrichment analysis (GSEA) was performed by the fgsea R package (v-1.12.0) (https://doi.org/10.1101/060012) with the MSigDB dataset (v-6.1)^37^ and was visualized by the “draw.GSEA” function of the NetBID software (v-2.0.2). Raw and processed RNA-seq data can be obtained in the GEO database under accession codes GSE149000 and GSE138437.

### qRT-PCR

An aliquot of 1.5 μg of total RNA was used to synthesize cDNA using a SuperScript VILO cDNA Synthesis Kit. Then, qPCR reactions for the genes DHFR, SHMT1, SHMT2, MTHFD2, TYMS, GLUT1, TXNIP, and GAPDH were performed using gene-specific primers (Table S2) and Fast SYBR Green Master Mix (Applied Biosystems) on an Applied Biosystems 7900HT Fast Real-Time PCR System (Life Technologies). Cycling conditions included denaturation (95 °C for 30 s), amplification and quantitation repeated for 40 cycles (95 °C for 5 s and 60 °C for 30 s, with a single fluorescence measurement), and a melting curve program to ensure that no primer dimers were formed. Gene expression levels were analyzed according to the comparative Ct method, using GAPDH as an internal control, and were calculated by the 2^−ΔΔCt^ method.

### Determination of glucose and lactate levels

The levels of cell glucose consumption and lactate secretion were determined with a Glucose-Glo Assay (Promega, J6021) and a Lactate-Glo Assay (Promega, J5021) according to the manufacturer's instructions. Briefly, supernatant from the culture medium was collected, centrifuged, and diluted with PBS. Then, 15 μL of each sample and 15 μL of glucose detection reagent or lactate detection reagent were added to a white 384-well plate (Corning, 8804BC) and mixed using a shaker for 1 min. The plate was incubated at RT for 60 min, and then the luminescence was read. The glucose and lactate concentrations were calculated according to the glucose and lactate standard curves. Glucose uptake was determined using a Glucose Uptake-Glo Assay (Promega, J1341). Before the assay was performed, the medium was removed from the cells, and they were washed twice with 2 mL of warm PBS. Next, 1 mL of the prepared 1 mM 2DG was added to each well, and the plate was shaken briefly and then incubated at RT for 10 min. After this incubation, 500 μL of stop buffer was added to each well and the plate was shaken briefly, and then 75 μL of each sample was transferred to a white 96-well plate. After the transfer, 25 μL of neutralization buffer was added to each well and the plate was shaken briefly, and then 100 μL of 2DG6P detection reagent was added and the plate was again shaken briefly. The plate was incubated at RT for 1 h, and then the luminescence was read. The 2DG6P concentration was calculated according to the 2DG6P standard curve.

### Cell surface protein biotinylation

For this part of the study, cells were grown in 6-well plates. After the cells reached adequate confluence, they were washed with warm PBS, and then 500 μL of biotinylation solution (1 mg/mL EX-link Maleimide-PEG2-Biotin; Thermo Scientific, 21901BID) and 1 mg/mL EZ-Link Sulfo-NHS-LC-LC-Biotin (Thermo Scientific, 21338) were added to each well and the plates were incubated at 37 °C for 20 min. The biotinylation solution was then removed, 1 mL of 100 mM glycine (in PBS) was added, and the plates were shaken gently to stop the reaction. The glycine buffer was then removed, and the cells were washed once with warm PBS. Next, 150 μL of RIPA buffer was added to lyse the cells. The cells were kept on ice for 20 min with shaking and then collected by scraping followed by centrifugation at 16,200 *g* at 4 °C for 20 min. The supernatant was collected, and the protein concentration was determined with a BCA assay. Next, the amounts of protein and monomeric avidin UltraLink Resin beads (Thermo Scientific, 53146) needed to perform the pull-down experiment were calculated, and these quantities were mixed (1 μg of protein per 1 μL of avidin beads). The mixture was rotated for 45 min on a 3D shaker. After the reaction, the mixture was centrifuged at 13,800 *g* at 4 °C for at least 1 min, and the supernatant (intracellular protein samples) was then carefully collected, taking care to avoid disturbing the beads. The mixture was centrifuged several times to collect as much supernatant as possible. The avidin beads were washed twice with 500 μL of PBS and then centrifuged at 13,800 *g* at 4 °C for 1 min. The PBS was initially removed with a 1-mL syringe via a 22G needle, and then the rest of the PBS was removed with a 10-μL tip. Laemmli buffer was added to the protein samples at a 2× concentration (Bio-Rad, 1610737EDU) for avidin beads binding protein and at a 4× concentration (Bio-Rad, 1610747) for intracellular protein. Briefly, the samples were centrifuged to mix the protein and Laemmli buffer and then boiled at 100 °C for 4 min. The avidin beads binding protein samples were then again centrifuged and the supernatants were collected. These are the surface protein samples.

### Immunofluorescence

For the siRNA reverse transfection, 0.55 pmol of siRNA was dispensed into a 384-well transparent-bottom plate (PerkinElmer, 6057508), using an Echo 555 liquid handler (Beckman). Then, 10 μL of 1% Lipofectamine RNAiMAX (Invitrogen, 10001447) in Opti-MEM medium (Gibco, 51985-034) was added to the siRNA. After a 20-min incubation, the cells were seeded in the plate. For the knockout cell lines, for which transfection is not needed, cells were seeded in the 384-well plate without prior transfection.

Cells in the 384-well plate were fixed by incubation with 4% paraformaldehyde for 15 min. Fixed cells were permeabilized by a 15-min incubation with 0.1% Triton X-100. Then, the cells were incubated at 4 °C overnight with Alexa Fluor 594 anti-GLUT1 antibody (Abcam, ab206360; diluted 1:100), Alexa Fluor 488 anti-ATP1A1 antibody (Abcam, ab197713; diluted 1:200), and 10 mg/mL Hoechst (diluted 1:2000) in 1% horse serum (Gibco, 26050070). After being washed with PBS, the cells were imaged with a Yokogawa CV8000 confocal microscopy–based imaging system fitted with a 60× objective lens.

Colocalization was quantified by CellProfiler software. Single cells were segregated as nuclear-stained images and ATP1A1-stained images using IdentifyPrimaryObjects and IdentifySecondaryObjects after smoothing the nuclear signal. Cells within a GLUT1 signal intensity threshold were selected for further analysis. For the colocalization analysis, Mander's coefficients were calculated using MeasureColocalization. For the statistical analysis, two groups (in the knockdown experiment) were analyzed using an unpaired *t*-test of correlation, and three groups (WT cells and the two types of CYP3A5-KO cells) were analyzed by one-way ANOVA.

### Determination of ROS levels

Cells were grown in culture in a white 96-well plate (Costar, 3916) and treated with different compounds as desired. The ROS levels were determined with a ROS-Glo H_2_O_2_ Assay (Promega, G8820) according to the manufacturer's instructions. Briefly, 20 μL of prepared H_2_O_2_ substrate solution was added to the cells and mixed, and the plate was placed in a 37 °C CO_2_ incubator for 5 h. Then, 100 μL of ROS-Glo™ Detection Solution was added to each well, and the plate was incubated at RT for 20 min, after which the relative luminescence units were recorded.

### Wound healing assay

AsPC-1 WT and CYP3A5-KO cells were seeded in a 6-well plate followed by adding siRNA the next day. Scratches were created by a 1 mL tip the next day when the wells are confluent. Then, the medium was discarded, and cells were washed with fresh medium to remove cell debris and covered with fresh medium (with the same or different glucose concentrations as indicated). Images of cells were taken by an Olympus CKX41 microscope (with an Infinity 1 camera) to examine wound healing.

### Organoid-like culture

AsPC-1 WT and AsPC-1 CYP3A5 KO cells were embedded in ECM (extracellular matrix, ATCC- ACS- 3035) (1000 cells per 1 mL ECM) supplemented with organoid media formulation #3 and cultured according to ATCC's organoid culture protocol (Organoid Subculture protocol). Images of the organoid-like culture were captured by an Olympus CKX41 microscope (with an Infinity 1 camera).

### Statistical analysis

The data were expressed as the mean ± standard deviation of at least three independent experiments, and statistical significance was established if the *P* value was less than 0.05. Student's *t*-test was used to compare the means of two groups as specified. Other analyses were performed using one-way ANOVA for all samples compared with the control or using a two-way ANOVA multiple comparison test in Graphpad Prism 9.0. ns, non-significant, *P* ≥ 0.05; ^∗^*P* < 0.05; ^∗∗^*P* < 0.01; ^∗∗∗^*P* < 0.001; ^∗∗∗∗^*P* < 0.0001.

## Results

### CYP3A5 is frequently up-regulated in pancreatic cancer

To investigate the role of CYP3A5 in cancer progression, we checked the level of CYP3A5 expression in different cancer cell lines based on the Cancer Cell Line Encyclopedia. This showed that cell lines derived from colorectal, kidney, stomach, pancreas, or bile duct cancers had higher CYP3A5 expression than did other cancer cell lines (Fig. S1A). To check whether CYP3A5 was involved in tumorigenesis, data from The Cancer Genome Atlas was analyzed to compare the difference in expression between tumor and matched normal samples for 24 primary cancer types. We found that in kidney renal papillary cell carcinoma, pancreatic adenocarcinoma, kidney renal clear cell carcinoma, glioblastoma multiforme, and skin cutaneous melanoma, CYP3A5 expression was higher in tumor tissues than in normal tissues (Fig. S1B). Among all the cancer cell lines examined, the PDAC cell line AsPC-1 had the highest expression of CYP3A5, while the normal pancreatic ductal epithelial cell line HPNE hardly expressed CYP3A5 (Fig. S1C). Furthermore, PDAC cells expressed little CYP3A4, a CYP3A5 analog, or other CYP3A gene products, which made PDAC cell lines, especially AsPC-1, good models for studying CYP3A5.^28^ Therefore, we chose the AsPC-1 and HPAF-II cell lines as our study models. Together, these data indicate that CYP3A5 is highly expressed in PDAC cells and may play an important role in PDAC progression.

### Metabolome profiling reveals that CYP3A5 knockdown alters glucose metabolism in AsPC-1 cells

**Figure 1:**
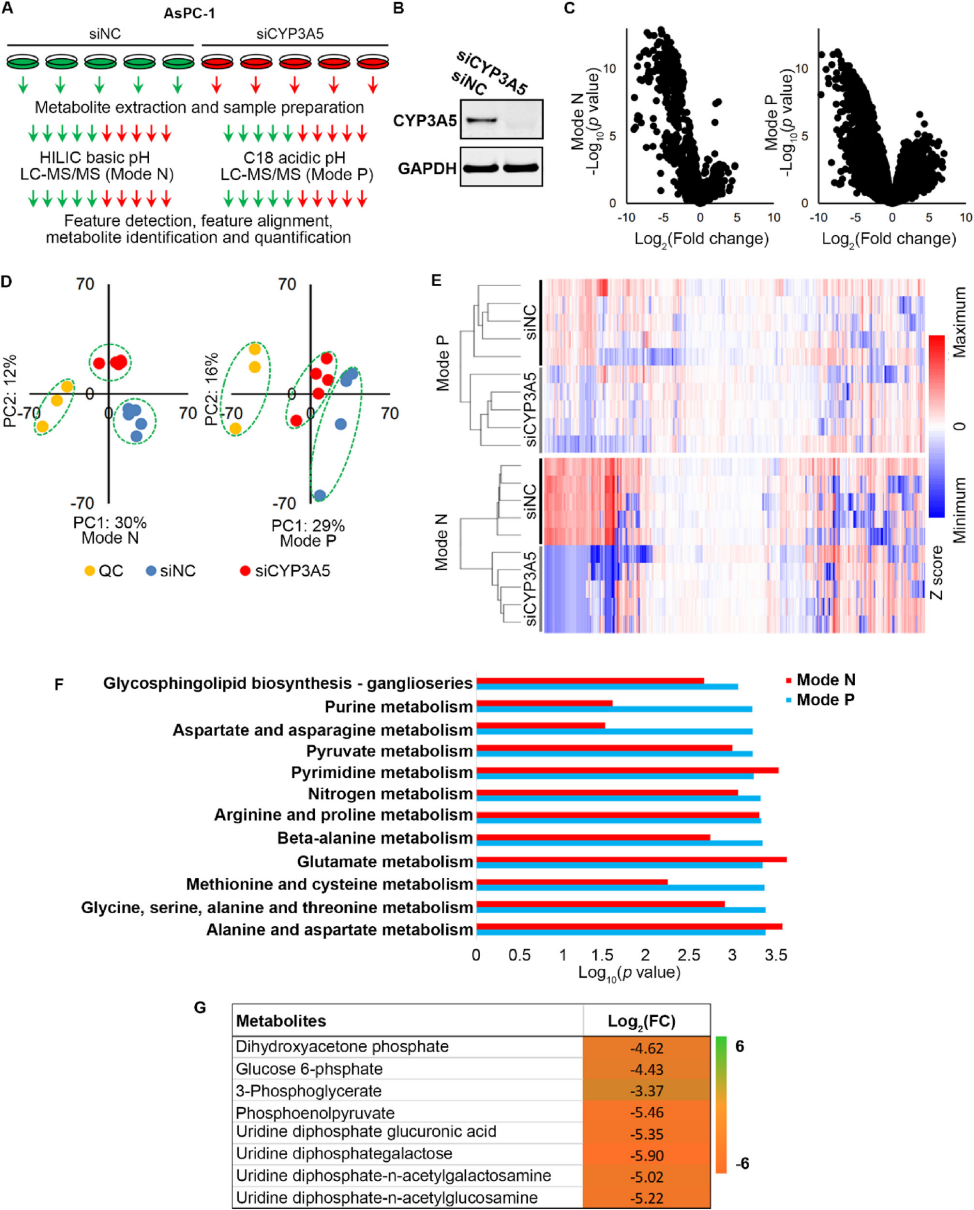
Metabolome profiling of CYP3A5-KD AsPC-1 cells. **(A)** Experimental scheme of metabolome profiling. **(B)** Immunoblots showing CYP3A5 levels in AsPC-1 cells after treatment with siNC or siCYP3A5. **(C)** Volcano plot of all detected features that changed significantly after CYP3A5 knockdown. **(D)** Principal component analysis of all detected features. QC samples are a mixture of 1/50 of all 10 samples. **(E)** Cluster analysis of all detected features. **(F)** Pathway analysis by MetaboAnalyst based on the feature list, with *P* < 0.005 as a cutoff. **(G)** List of the glucose metabolism-related metabolites that changed significantly (FC > 2, *P* < 0.05) after CYP3A5 knockdown.

Since CYP3A5 protein is an enzyme, we performed a metabolome profiling analysis of AsPC-1 cells after CYP3A5 knockdown to assess the metabolic changes as a first step toward determining the role of CYP3A5 in PDAC. AsPC-1 cells were treated with siNC or siCYP3A5 for 3 days before the metabolites were extracted. The extracted metabolites were analyzed by hydrophilic interaction liquid chromatography LC–MS/MS (in negative mode) and C18-LC–MS/MS (in positive mode), respectively. After the metabolites were identified and quantified, their features were detected and aligned (Fig. 1A). The efficiency of CYP3A5 knockdown by siCYP3A5 was confirmed by Western blot analysis, which showed that the CYP3A5 protein band was undetectable after siCYP3A5 treatment (Fig. 1B). Interestingly, as shown in the volcano plot in Figure 1C, CYP3A5 knockdown induced significant metabolomic changes, mainly down-regulation of metabolites, as reflected in data obtained in both positive mode (741 of 4983 features) and negative mode (294 of 1149 features, fold change > 2, *P* < 0.05). Approximately 40% of the features in data from the positive mode and 25% of the features in data from the negative mode had putative structure IDs with level 2 confidence (when matched to a database or the literature). Principal component analysis (Fig. 1D) and clustering analysis (Fig. 1E) of all the detected features further verified the reproducibility of these results. Pathway analysis showed that the metabolic pathways that were changed after CYP3A5 knockdown included purine metabolism, pyruvate metabolism, and glycine, serine, alanine, and threonine metabolism, among others (Fig. 1F). Therefore, CYP3A5 clearly played an important role in AsPC-1 metabolism. To better identify which metabolites were changed, we performed a level 1 ID validation using a reference standard match based on our in-house compound library. A volcano plot indicated that metabolites were down-regulated significantly in CYP3A5-KD cells (Fig. S2A). Principal component analysis (Fig. S2B) and clustering analysis (Fig. S2C) of all identified and quantified metabolites verified the reproducibility of these results. The changed metabolites included glucose-derived metabolites, nucleotides, and tricarboxylic acid cycle-related metabolites (Fig. 1G; Fig. S2D). Key metabolites in the glycolysis pathway, such as glucose-6-phosphate, 3-phosphoglycerate, and phosphoenolpyruvate, were robustly decreased, as were some hexosamine-related metabolites (Fig. 1G). In summary, the above results indicated that CYP3A5 knockdown altered AsPC-1 glucose metabolism.

### CYP3A5 down-regulation decreases glucose uptake in AsPC-1 cells

**Figure 2:**
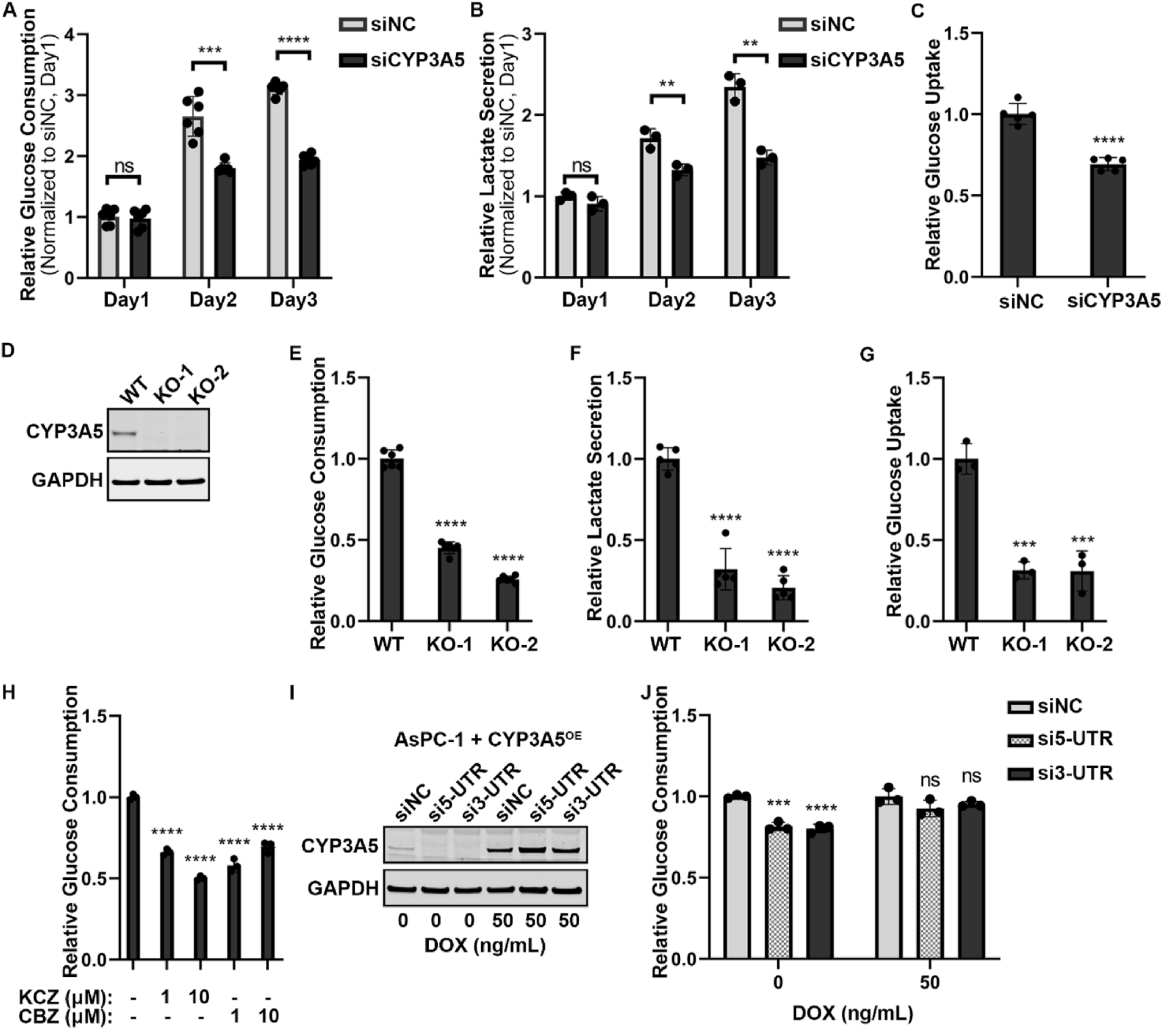
CYP3A5 down-regulation results in decreased glucose uptake. **(A)** Glucose consumption of AsPC-1 cells after treatment with siNC or siCYP3A5 siRNA. **(B)** Lactate secretion of AsPC-1 cells after treatment with siNC or siCYP3A5. **(C)** Glucose uptake of AsPC-1 cells after treatment with siNC or siCYP3A5. **(D)** Immunoblots showing the levels of CYP3A5 in AsPC-1 wild-type (WT) and CYP3A5-deletion cell lines (KO-1 and KO-2). **(E)** Glucose consumption of AsPC-1 WT and CYP3A5-KO cells. **(F)** Lactate secretion of AsPC-1 and CYP3A5-KO cells. **(G)** Glucose uptake of AsPC-1 WT and CYP3A5-KO cells. **(H)** Glucose consumption of AsPC-1 cells after treatment with the CYP3A5 inhibitors ketoconazole (KCZ) and clobetasol propionate (CBZ) (at 1 μM and 10 μM for 2 days). **(I)** Immunoblots showing the levels of CYP3A5 after CYP3A5 was overexpressed (in the presence of 50 ng/mL DOX) in AsPC-1 cells (AsPC-1 CYP3A5-OE cells) treated with siNC or siCYP3A5s targeting the UTR region of the CYP3A5 gene (si5-UTR and si3-TUR). **(J)** Glucose consumption of AsPC-1 CYP3A5-OE cells after the CYP3A5 level was rescued in the control and CYP3A5-KD groups.

To confirm further that CYP3A5 regulated glucose metabolism in AsPC-1 cells, we first checked the glycolysis change after CYP3A5 down-regulation. After treating cells with siCYP3A5 for 3 days, the medium was collected to determine the glucose and lactate concentrations. The glucose consumption was calculated from the original and final glucose concentrations in the medium. This showed that CYP3A5 knockdown resulted in a significant decrease in glucose consumption (Fig. 2A) and lactate secretion (Fig. 2B). The change in glucose uptake was further determined by direct measurement with the Glucose Uptake-Glo Assay. 2DG was used to replace the medium that contained glucose, and the glucose uptake was determined by measuring the concentration of 2DG6P, which was converted from 2DG by the cells. The glucose uptake of the siCYP3A5-treated group was also decreased to only 70% of that of the control group (Fig. 2C). The individual siCYP3A5 siRNAs were applied separately to ensure that the result obtained with the siCYP3A5 pool was reliable. All three siCYP3A5 siRNAs decreased CYP3A5 protein levels (Fig. S3A, C) and reduced glucose consumption in both AsPC-1 cells and HPAF-II cells (Fig. S3B, D). We next examined the glucose uptake in AsPC-1 CYP3A5-deletion cell lines. The CYP3A5 protein was undetectable in the two AsPC-1 CYP3A5-deletion cell clones (KO-1 and KO-2) (Fig. 2D). The glucose consumption (Fig. 2E), lactate secretion (Fig. 2F), and glucose uptake (Fig. 2G) were all significantly decreased in both CYP3A5-KO cells. We then used two CYP3A5 inhibitors to see whether inhibition of CYP3A5 function by compounds could mimic the effect of CYP3A5 knockdown/knockout on cell glucose uptake. Ketoconazole is a gold-standard pan-CYP3A inhibitor,^38^ and clobetasol propionate is a selective CYP3A5 inhibitor.^28^ Cells were treated with 1 μM or 10 μM preparations of inhibitors (in a final DMSO concentration of 0.1%) for 2 days, and then the medium was collected for glucose assay. This showed that treatment with a CYP3A5 inhibitor significantly decreased glucose consumption, just as CYP3A5 knockdown/knockout did (Fig. 2H). In the last step of this experiment, we overexpressed CYP3A5 when CYP3A5 was knocked down by siCYP3A5 and investigated whether this could rescue glucose uptake. AsPC-1 cells with inducible CYP3A5 were placed in a culture with 20 ng/mL doxycycline (DOX) to induce the CYP3A5 protein. siCYP3A5-5UTR (si5-UTR) and siCYP3A5-3UTR (si3-UTR) could target the endogenous *CYP3A5* mRNA but could not decrease the induced ectopic *CYP3A5* mRNA. This showed that in the absence of induced CYP3A5 protein, si-5UTR and si-3UTR could decrease CYP3A5 protein (Fig. 2I) and reduce glucose consumption (Fig. 2J). After DOX was added to induce the CYP3A5 protein (Fig. 2I), the glucose consumption recovered (Fig. 2J). In summary, these findings prove that CYP3A5 regulates glucose uptake in pancreatic cancer cells.

### CYP3A5 knockdown affects glucose transport

To our knowledge, this is the first report that CYP3A5 can affect glucose uptake in tumor cells. To determine how CYP3A5 down-regulation causes glycolytic changes in AsPC-1 cells, we performed RNA-seq analysis to investigate the transcriptome changes upon CYP3A5 knockdown. For each sample, we generated more than 40 million high-quality reads (Fig. S4A). The gene body coverage analysis showed that the RNAs extracted from each sample were of high integrity (Fig. S4B). We employed two different methods to quantify the expression of genes, and the results of these two methods overlapped considerably with an R2 greater than 0.92 (Fig. S4C), showing the very high accuracy of the quantification results obtained in this study.

**Figure 3:**
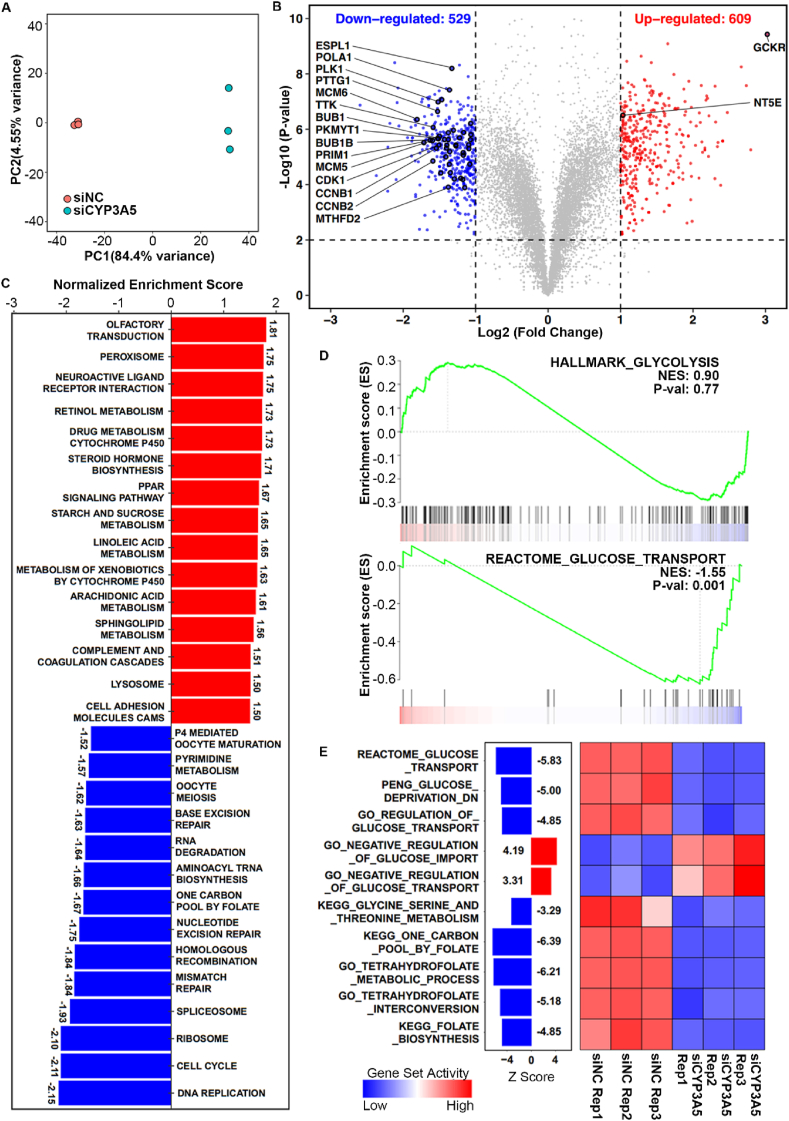
Transcriptomic analyses of CYP3A5 knockdown in AsPC-1 cells. **(A)** Principal component analysis plot of all six samples using the stats R package (v3.6.1) (*n* = 3 for each group). **(B)** Volcano plot of gene expression changes between CYP3A5-knockdown (KD) and wild-type (WT) samples. The red dots indicate genes up-regulated in CYP3A5-KD samples (*P* < 0.01 and log_2_ FC > 1), and the blue dots indicate genes down-regulated in CYP3A5-KD samples (*P* < 0.01 and log_2_ FC < −1). Genes involved in KEGG_CELL_CYCLE, KEGG_ONE_CARBON_POOL_BY_FOLATE, KEGG_PYRIMIDINE_METABOLISM, and REACTOME_GLUCOSE_TRANSPORT are highlighted with a black stroke, and the top-ranked ones are annotated and marked. *P* values were determined by the limma package. *n* = 3 for each group. **(C)** Bar plot showing the results of gene set enrichment analysis (GSEA) of KEGG pathways, using a gene list pre-ranked by fold change in gene expression between CYP3A5-KD and WT samples. The significantly enriched gene sets were identified (*P* < 0.01) and further ranked by normalized enrichment score. The red bars indicate the KEGG pathways activated, and the blue bars indicate those pathways suppressed upon CYP3A5 knockdown in AsPC-1 cells. **(D)** GSEA plots of selected gene sets generated by the draw.GSEA function of NetBID. The normalized enrichment scores and *P* values were calculated by fgsea. **(E)** Heatmap showing the change in gene set activity between CYP3A5-KD and WT samples. The gene sets related to glucose uptake, amino acid metabolism, and folate metabolism were selected. The activity of the gene sets was inferred by NetBID, and the Z-scores were calculated by limma.

With the high-quality RNA-seq data, we observed a high similarity among the intra-group samples and a clear separation between groups with and without CYP3A5 knockdown (Fig. 3A). Consistently, the differential analysis of these two groups revealed an extensive transcriptomic change upon CYP3A5 knockdown, with 609 genes being up-regulated and 529 down-regulated under the cutoffs of *P* < 0.01 and fold change > 2 (Fig. 3B). We further explored the pathway perturbation upon CYP3A5 knockdown using GSEA. We identified a total of 29 significantly enriched pathways from the Kyoto Encyclopedia of Genes and Genomes (KEGG) dataset under the cutoff of *P* < 0.01, including 15 up-regulated pathways and 14 down-regulated pathways (Fig. 3C). The up-regulated pathways that were a few related to *CYP*-family genes included retinol metabolism, drug metabolism via cytochrome P450, and steroid hormone biosynthesis. This indicated that some other *CYP* genes might be up-regulated upon CYP3A5 knockdown. Intriguingly, among the downregulated pathways, we identified some were highly associated with the decrease in glucose uptake, including pyrimidine metabolism, one-carbon pool metabolism by folate, and the cell cycle (Fig. 3C). Based on metabolome profiling analysis, glucose metabolism, purine/pyrimidine metabolism, and glycine/serine metabolism changed after CYP3A5 knockdown. The *de novo* biosynthesis of serine started from glucose, and serine could enter the folate cycle, which also produced glycine.^26^^,^^39^ Both the folate cycle and glucose could be used to generate nucleotides, which are important for DNA synthesis.^24^^,^^40^, ^41^, ^42^ The transcriptome results were highly consistent with those that we generated from metabolome data. We also found that genes involved in glucose transport, but not in glycolysis, were down-regulated upon CYP3A5 knockdown (Fig. 3D). To confirm our observations with GSEA, we employed a systems biology method that could estimate the activity of gene sets by calculating the average expression of the genes involved.^35^ The new algorithm provided results consistent with those of the GSEA, showing that the pathways related to glucose transport, glycine/serine metabolism, and folate-THF metabolism went down upon CYP3A5 knockdown (Fig. 3E).

### CYP3A5 enriches GLUT1 at the plasma membrane

**Figure 4:**
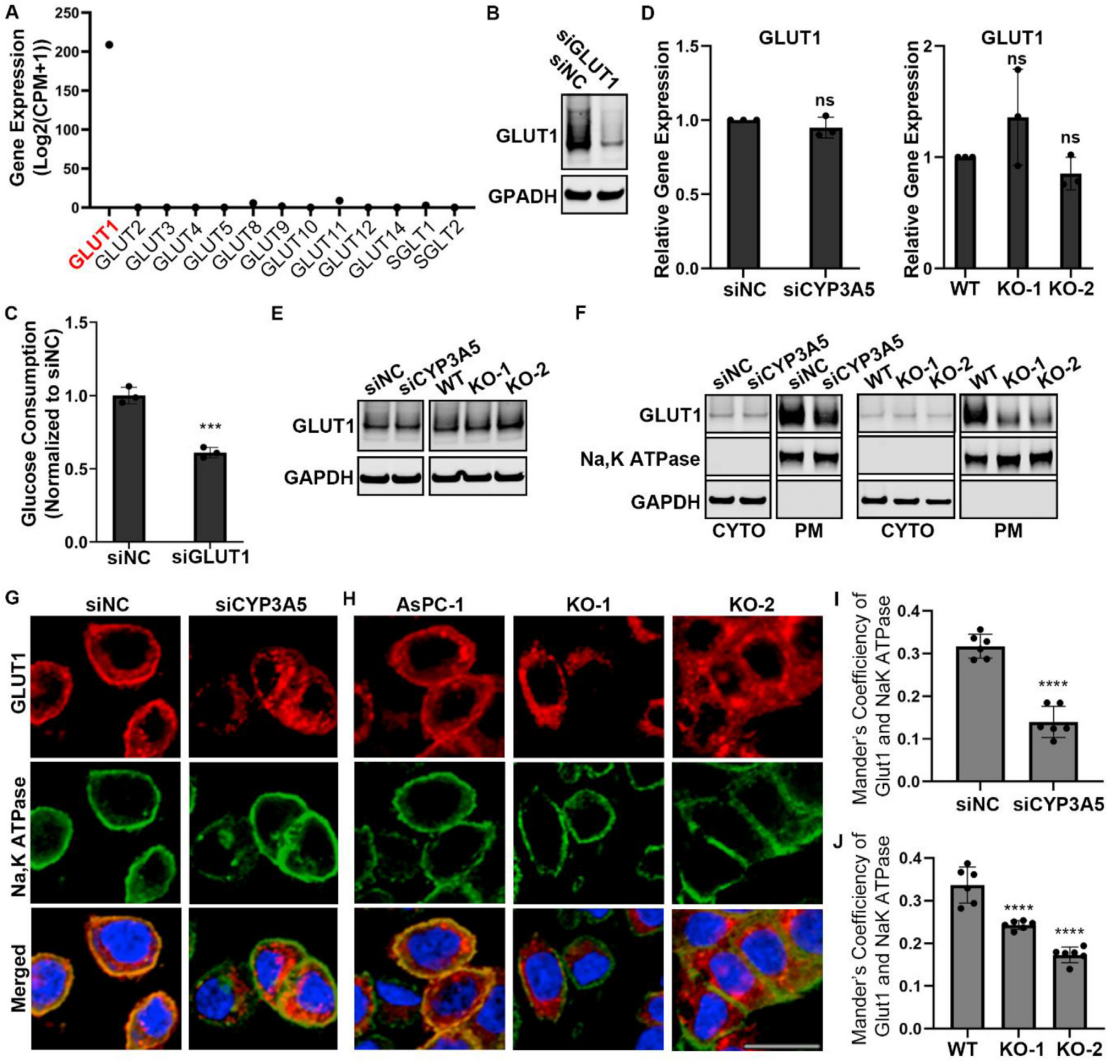
CYP3A5 affects membrane translocation of GLUT1 instead of its mRNA or protein level. **(A)** Expression levels of various glucose transporters in AsPC-1 cells, based on RNA-seq data. **(B)** Immunoblots showing the levels of GLUT1 in AsPC-1 cells after siGLUT1 treatment. **(C)** Glucose consumption of AsPC-1 cells after siGLUT1 treatment. **(D)** qPCR analysis of GLUT1 mRNA levels in AsPC-1 cells after CYP3A5 down-regulation. **(E)** Immunoblots showing the levels of total GLUT1 in AsPC-1 cells after CYP3A5 down-regulation. **(F)** Immunoblots showing the levels of cytosol-localized and plasma membrane-localized GLUT1 in AsPC-1 cells after CYP3A5 down-regulation. **(G, H)** Immunofluorescence of GLUT1 (red), NaK ATPase (green), and nuclei (blue) in AsPC-1 cells after CYP3A5 knockdown (G) or knockout (H). Scale bar = 20 μm. **(I, J)** Quantification of plasma membrane-localized GLUT1, based on colocalization with a plasma membrane protein (NaK ATPase), as in the immunofluorescence images in panels G and H.

Inspired by the results of integrative analysis of metabolome and transcriptome profiling, we examined whether CYP3A5 regulated glucose transporters. Based on the RNA-seq data, SLC2A1 (GLUT1) was the dominant glucose transporter among the different glucose transporter isoforms in AsPC-1 and HPAF-II cells (Fig. 4A; Fig. S5C). GLUT1 was up-regulated in many cancer cells and had a wide tissue distribution.^43^, ^44^, ^45^ We then investigated whether GLUT1 was responsible for glucose uptake in AsPC-1 cells. After cells were treated with siGLUT1 for 3 days, the GLUT1 protein level was decreased (Fig. 4B; Fig. S5A, D), as was the glucose consumption of the cells (Fig. 4C; Fig. S5B, E). We then examined the GLUT1 level change after CYP3A5 down-regulation. qPCR data showed that the level of GLUT1-encoding mRNA was not significantly decreased after CYP3A5 knockdown/knockout (Fig. 4D; Fig. S5F), and Western blot analysis showed that the GLUT1 protein level was not decreased either (Fig. 4E).

In addition to transcriptional, translational, and post-translational regulation of GLUT1,^46^, ^47^, ^48^ the membrane translocation of the protein from the cytosol to the plasma membrane is also important for GLUT1 regulation and is regulated by the cells.^43^^,^^49^^,^^50^ GLUT1 must be localized at the plasma membrane to be functional for transporting glucose. Therefore, we investigated whether there was any change in the subcellular distribution of GLUT1. Cells were incubated with a biotinylation solution before being harvested, and cytosol proteins and membrane proteins were separated by avidin UltraLink resin beads. This showed that after CYP3A5 knockdown/knockout, the GLUT1 protein level in the cytosol was not decreased, whereas the plasma membrane-localized GLUT1 was clearly decreased (Fig. 4F; Fig. S5G). This was further confirmed by a GLUT1 immunofluorescence study (Fig. 4G and H). There was less GLUT1 (red) localized to the plasma membrane (green, represented by the plasma membrane protein NaK ATPase) in CYP3A5-KD cells (Fig. 4G) and CYP3A5-KO cells (Fig. 4H). We quantified the colocalization of GLUT1 and NaK ATPase to measure the localization of GLUT1 in the plasma membrane and found that this localization was reduced significantly in the CYP3A5-KD/KO groups (Fig. 4I, J). In summary, CYP3A5 enriched GLUT1 at the plasma membrane.

### CYP3A5 regulates the translation of TXNIP, a negative regulator of GLUT1

**Figure 5:**
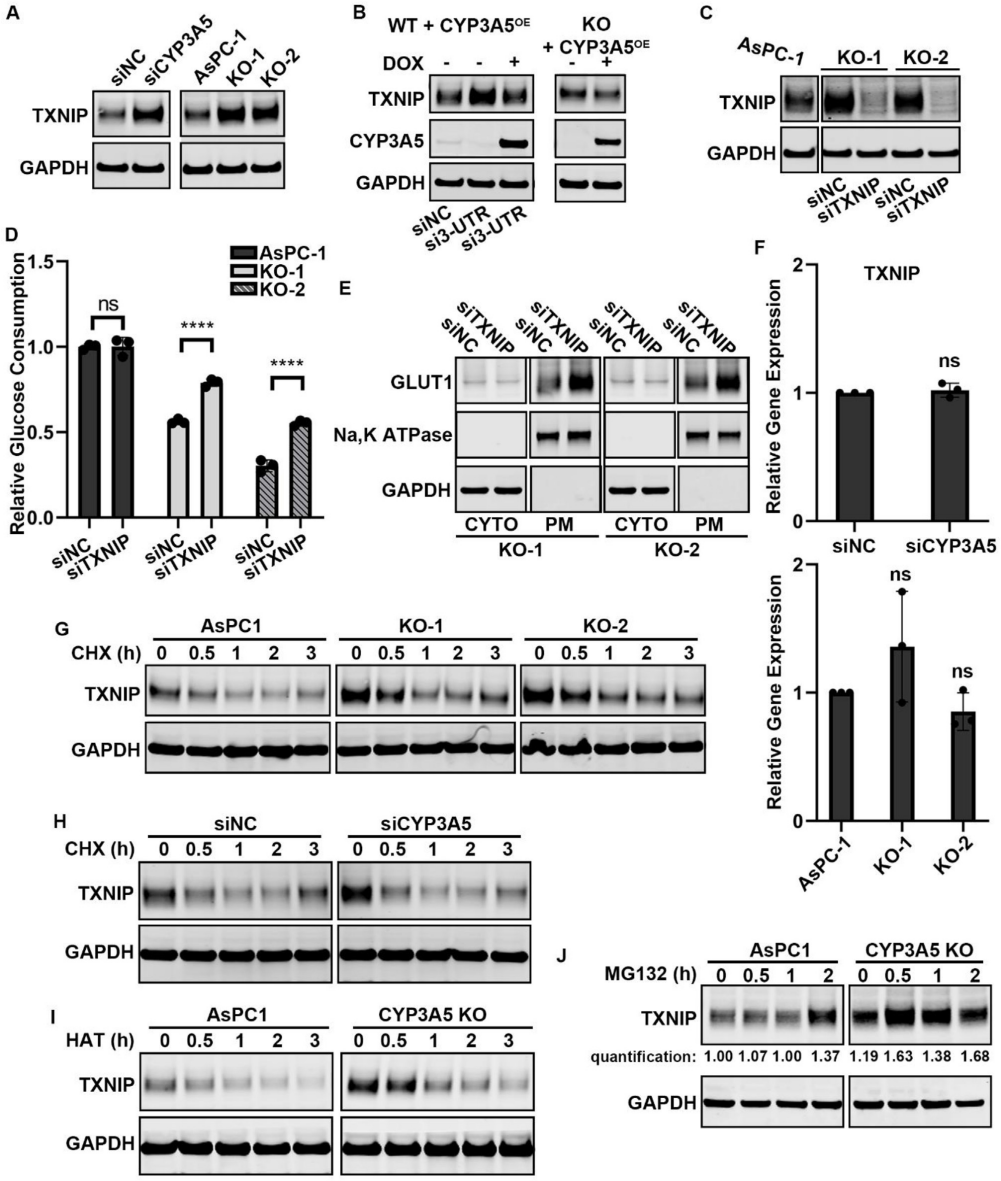
CYP3A5 enhances membrane translocation of GLUT1 by decreasing TXNIP translation. **(A)** Immunoblots showing the levels of TXNIP in AsPC-1 cells after CYP3A5 knockdown/knockout. **(B)** Immunoblots showing the levels of TXNIP in CYP3A5-KD/KO cells after CYP3A5 overexpression was induced by treatment with DOX (50 ng/mL). **(C)** Immunoblots showing the levels of TXNIP in CYP3A5-KO cells after siTXNIP treatment. **(D)** Glucose consumption of CYP3A5-KO cell lines after they were treated with siTXNIP for 3 days. **(E)** Immunoblots showing the levels of cytosol-localized and plasma membrane-localized GLUT1 in CYP3A5-KO cell lines after treatment with siTXNIP for 3 days. **(F)** qPCR analysis of *TXNIP* mRNA levels after CYP3A5 down-regulation. **(G)** Immunoblots showing the levels of TXNIP in WT and CYP3A5-KO cells after treatment with CHX (10 μM). **(H)** Immunoblots showing the levels of TXNIP in siNC and siCYP3A5 cells after treatment with CHX (10 μM). **(I)** Immunoblots showing the levels of TXNIP in WT and CYP3A5-KO cells after treatment with harringtonine (10 μM). **(J)** Immunoblots showing the levels of TXNIP in WT and CYP3A5-KO cells after treatment with MG132 (10 μM).

We next sought to determine how CYP3A5 affected GLUT1 translocation. One of the mediators involved in regulating GLUT1 translocation is TXNIP (thioredoxin interacting protein), a multifunctional regulator that promotes GLUT1 internalization.^43^^,^^50^^,^^51^ We found that TXNIP was regulated by CYP3A5 in our study models. After CYP3A5 knockdown/knockout, the TXNIP protein level was clearly increased (Fig. 5A; Fig. S6A). To confirm further that CYP3A5 regulated TXNIP level, we overexpressed CYP3A5 in CYP3A5-KD/KO groups and investigated whether the increased TXNIP protein level could be reduced. After siCYP3A5 (si3-UTR) treatment of AsPC-1 cells, the TXNIP protein level was up-regulated. Then, when cells were treated with DOX to induce CYP3A5 expression, the TXNIP protein level was down-regulated to that in the siNC control group (Fig. 5B). A similar result was obtained with the AsPC-1 CYP3A5-KO cell line. Overexpression of CYP3A5 in CYP3A5-KO cells decreased the TXNIP protein level (Fig. 5B). To examine whether TXNIP was related to glucose uptake in AsPC-1 cells, we knocked down TXNIP in AsPC-1 CYP3A5-KO cell lines and determined the change in glucose uptake. After CYP3A5-KO cells were treated with siTXNIP for 3 days, the TXNIP protein level was drastically decreased (Fig. 5C) and glucose uptake recovered (Fig. 5D). This result indicated that TXNIP was a negative regulator of glucose uptake in AsPC-1 cells. We then investigated whether the recovery of glucose uptake in CYP3A5-deletion cell lines after TXNIP knockdown was due to a change in the location of GLUT1. After CYP3A5-KO cells were treated with siTXNIP, there was little change in the cytosol GLUT1, whereas the plasma membrane-localized GLUT1 was clearly increased (Fig. 5E). This result indicated that TXNIP regulated glucose uptake by controlling GLUT1 membrane translocation.

Studies of TXNIP regulation have usually focused on two regulatory mechanisms: transcription and protein degradation.^43^^,^^50^^,^^51^ To investigate further how CYP3A5 regulated the TXNIP level, we first examined whether it was a transcription-related process. We used qPCR to measure the level of *TXNIP* mRNA after CYP3A5 knockdown/knockout and found no significant increase in *TXNIP* mRNA with either form of CYP3A5 down-regulation (Fig. 5F; Fig. S6BC). We then examined whether the regulation was accomplished through a change in protein degradation. Cycloheximide (CHX), the most common translation inhibitor, was used to inhibit new protein synthesis so that we could assess the change in pre-existing protein after CYP3A5 knockdown/knockout. This experiment showed that the amount of TXNIP protein decreased quickly in CYP3A5-KO cells (Fig. 5G) and CYP3A5-KD cells (Fig. 5H; Fig. S6C) after CHX treatment. Thus, CYP3A5 down-regulation did not stabilize TXNIP. Therefore, a change in protein degradation was not the reason why there was more TXNIP protein in CYP3A5-KO/KD cells. Another translational protein synthesis inhibitor, harringtonine, was also applied with a similar result, with TXNIP protein being degraded quickly in CYP3A5-KO cells (Fig. 5I).

Because the increased TXNIP protein level after CYP3A5 knockdown/knockout was not due to a change in transcription or protein degradation, we next investigated whether it resulted from a translation event. MG132, a widely used proteasome inhibitor, was applied to inhibit protein degradation so that we could measure the change in new protein. After CYP3A5-KO cells were treated with MG132, the TXNIP protein level increased more quickly, even though the basal TXNIP level in the CYP3A5-KO cells was already much higher than that in WT cells (Fig. 5J; Fig. S6D). This indicated that CYP3A5 knockout resulted in increased translation of TXNIP. Taken together, these findings showed that CYP3A5 inhibited the translation of TXNIP, a regulator that decreases the enrichment of GLUT1 at the plasma membrane.

### CYP3A5-generated ROS are responsible for the AKT–4EBP1–TXNIP axis regulation

**Figure 6:**
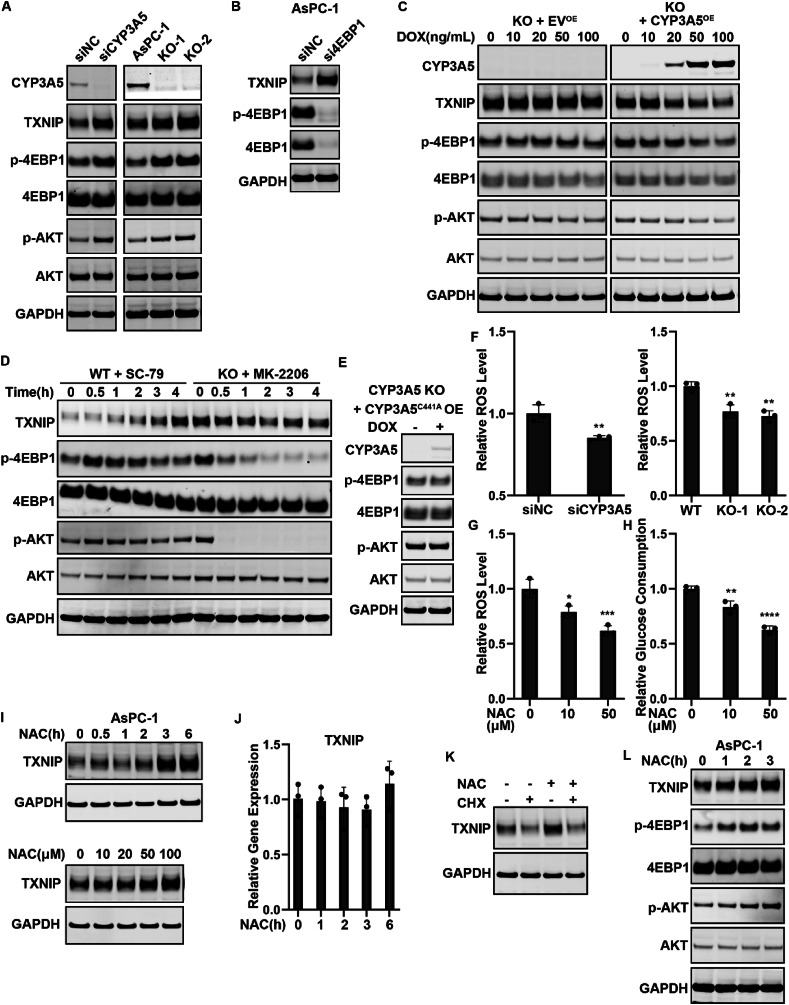
CYP3A5-generated ROS are responsible for AKT–4EBP1–TXNIP axis regulation. **(A)** Immunoblots showing the levels of CYP3A5, TXNIP, p-4EBP1, and p-AKT in AsPC-1 cells after CYP3A5 knockdown/knockout. **(B)** Immunoblots showing the levels of TXNIP, p-4EBP1, and 4EBP1 in AsPC-1 cells after si4EBP1 treatment. **(C)** Immunoblots showing the levels of CYP3A5, TXNIP, p-4EBP1, and p-AKT in AsPC-1 CYP3A5-KO cells after CYP3A5 overexpression was induced by DOX (ng/mL). **(D)** Western blot of TXNIP, p-4EBP1, and p-AKT in WT cells after treatment with SC-79 (10 μM) and in CYP3A5-KO cells treated with MK-2206 (10 μM). **(E)** Immunoblots showing the levels of CYP3A5, p-4EBP1, and p-AKT in CYP3A5-KO cells after overexpression of CYP3A5^C411A^. **(F)** Measurement of the ROS level in CYP3A5-KD/KO cells. **(G, H)** Measurement of the changes in the ROS level (G) and glucose consumption level (H) after treatment with NAC (μM). **(I)** NAC affected the TXNIP protein level in a time-dependent manner (when the concentration of NAC was 50 μM) and in a dose-dependent way (when the cells were treated for 3 h). **(J)** qPCR of TXNIP mRNA after cells were treated with NAC (50 μM). **(K)** Immunoblots showing the levels of TXNIP after treatment with NAC (50 μM), CHX (10 μM), and both. **(L)** Immunoblots showing the levels of TXNIP, p-4EBP1, and p-AKT in AsPC-1 cells after treatment with NAC (50 μM).

We next asked which upstream events might culminate in the reduction of TXNIP translation after CYP3A5 down-regulation. Protein synthesis consists of four steps: initiation, elongation, termination, and recycling.^52^, ^53^, ^54^ Initiation is the rate-limiting step in mRNA translation. 4EBP1 (EIF4EBP1) could bind to the translation initiation factor eIF4E to inhibit cap-dependent translation. Hyperphosphorylation of 4EBP1 disrupted this interaction and resulted in the activation of translation.^55^^,^^56^ We found that after knocking down or knocking out CYP3A5, the amount of phosphorylated 4EBP1 was clearly increased (Fig. 6A; Fig. S7C). CYP3A5 decreased 4EBP1 phosphorylation, thus reducing TXNIP translation. Silencing 4EBP1 expression with si4EBP1 prevented the inhibitory effect of CYP3A5 on TXNIP translation (Fig. 6B; Fig. S7D), further indicating that the ability of CYP3A5 to decrease TXNIP translation was 4EBP1 dependent.

With regard to how CYP3A5 regulated 4EBP1 phosphorylation, we examined the upstream regulators known to regulate 4EBP1 phosphorylation. The PI3K–AKT–mTOR signaling pathway promotes 4EBP1 phosphorylation and contributes to the release of 4EBP1 from eIF4E.^56^ We found that CYP3A5 down-regulation resulted in a clear increase in phosphorylated AKT (Fig. 6A; Fig. S7C). This might explain how CYP3A5 affects 4EBP1 phosphorylation. To confirm further that CYP3A5 regulated AKT phosphorylation and 4EBP1 phosphorylation, we overexpressed CYP3A5 in CYP3A5-deletion AsPC-1 cells. This showed that CYP3A5 overexpression could decrease the levels of p-AKT and p-4EBP1 in CYP3A5-KO cells, whereas there was no such change in the empty vector (EV) control group (Fig. 6C). These results indicated that CYP3A5 regulated AKT phosphorylation. To obtain further proof that changes in AKT phosphorylation resulted in the changes in p-4EBP1, we treated WT cells with SC-79, an AKT activator, treated CYP3A5-KO cells with MK-2206, an AKT inhibitor, and measured the change in the protein levels of TXNIP, p-AKT, and p-4EBP1. The p-AKT and p-4EBP1 levels were increased after the first few hours of treatment with the AKT activator, as was the TXNIP protein level. In CYP3A5-KO cells, the p-AKT protein had disappeared after MK-2206 treatment, and the p-4EBP1 protein and the TXNIP protein levels were decreased (Fig. 6D; Fig. S7E). These results suggested that CYP3A5 could inhibit AKT, thus decreasing 4EBP1 phosphorylation.

To elucidate the mechanism by which CYP3A5 regulated AKT phosphorylation, we first investigated whether CYP3A5 regulated AKT-4EBP1-TXNIP axis through its enzymatic function. We constructed a mutant CYP3A5 that had lost its enzymatic function (Fig. S7A, B). Overexpressing the CYP3A5^C411A^ did not change the level of p-AKT or p-4EBP1 (Fig. 6E), indicating that CYP3A5 regulated AKT via its enzymatic function. CYP enzymes catalyze the oxygenation of an organic substrate and the simultaneous reduction of molecular oxygen. If the transfer of oxygen to a substrate is not tightly controlled, uncoupling occurs and leads to the formation of ROS.^57^, ^58^, ^59^ Therefore, we examined whether ROS–AKT signaling was involved in the regulation of 4EBP1 phosphorylation by CYP3A5. As it was unknown whether CYP3A5 was related to ROS generation in PDAC, we first investigated whether CYP3A5 contributed to producing ROS in PDAC cells. A ROS assay showed that the ROS levels in cells were significantly decreased after CYP3A5 siRNA treatment or CYP3A5 deletion (Fig. 6F; Fig. S7F). We treated WT cells with the antioxidant N-acetyl cysteine (NAC) to decrease ROS (Fig. 6G; Fig. S7G) and found that glucose consumption was also decreased (Fig. 6H; Fig. S7H). We then examined whether the AKT–4EBP1–TXNIP pathway was a downstream target of ROS. NAC showed a dose-dependent and time-dependent effect in regulating the TXNIP protein level (Fig. 6I). NAC treatment did not affect the level of *TXNIP* mRNA (Fig. 6J), nor did it affect the degradation of TXNIP protein, as co-treatment with NAC and CHX decreased the TXNIP protein level (Fig. 6K). This result indicated that ROS could regulate TXNIP translation. We further examined the change in p-AKT and p-4EBP1. After cells were treated with NAC, p-AKT was clearly increased, as was p-4EBP1 (Fig. 6L; Fig. S7I). These results indicated that CYP3A5-generated ROS contributed to the AKT–4EBP1–TXNIP signaling pathway regulation.

### Altered glucose metabolism caused by CYP3A5 down-regulation decreases cell migration

After revealing the mechanism by which CYP3A5 affected glucose metabolism, we investigated the functional consequences of the decreased glucose metabolism caused by CYP3A5 down-regulation. To examine the effect on cell growth, based on changes in cell confluence, we seeded WT and CYP3A5-KO AsPC-1 cells at a low initial confluence. Interestingly, CYP3A5 knockout did not decrease cell growth (Fig. S8A). Given that the cells were grown in rich RPMI 1640 medium, it is likely that they could use another nutrient to compensate for the reduced glucose uptake. According to the RNA-seq data, both GSEA and gene set activity analysis showed that one metabolic pathway that was decreased after CYP3A5 knockdown was the serine/glycine and folate cycle pathway. qPCR assays confirmed that the genes related to the serine/glycine and folate cycle were indeed decreased after CYP3A5 knockdown (Fig. S8B). The protein levels of several key enzymes were also measured, which revealed that the levels of DHFR, PSAT1, and SHMT2 proteins were all decreased (Fig. S8C). The decreased glucose uptake affected the *de novo* synthesis of serine/glycine, which are also precursors for the one carbon-dependent folate cycle. We hypothesized that the cells could use exogenous serine/glycine when the synthesis of serine/glycine was affected by decreased glucose uptake. To confirm this, we grew AsPC-1 cells in a medium that lacked serine/glycine and found that CYP3A5 knockout clearly decreased cell growth under this condition (Fig. S8D). CYP3A5 still regulated glucose uptake in the AsPC-1 cells in the serine/glycine-limited condition and knocking down TXNIP resulted in a significant recovery of glucose uptake in AsPC-1 CYP3A5-KO cells (Fig. S8E). To confirm further that the cell growth defect was due to the glucose uptake decrease in CYP3A5-KO cells, we examined the effect on cell growth of decreasing TXNIP in CYP3A5-KO cells. We found that the growth of CYP3A5-KO cells recovered after siTXNIP treatment (Fig. S8F). Therefore, decreased glucose uptake made AsPC-1 cells more dependent on exogenous serine/glycine.

**Figure 7:**
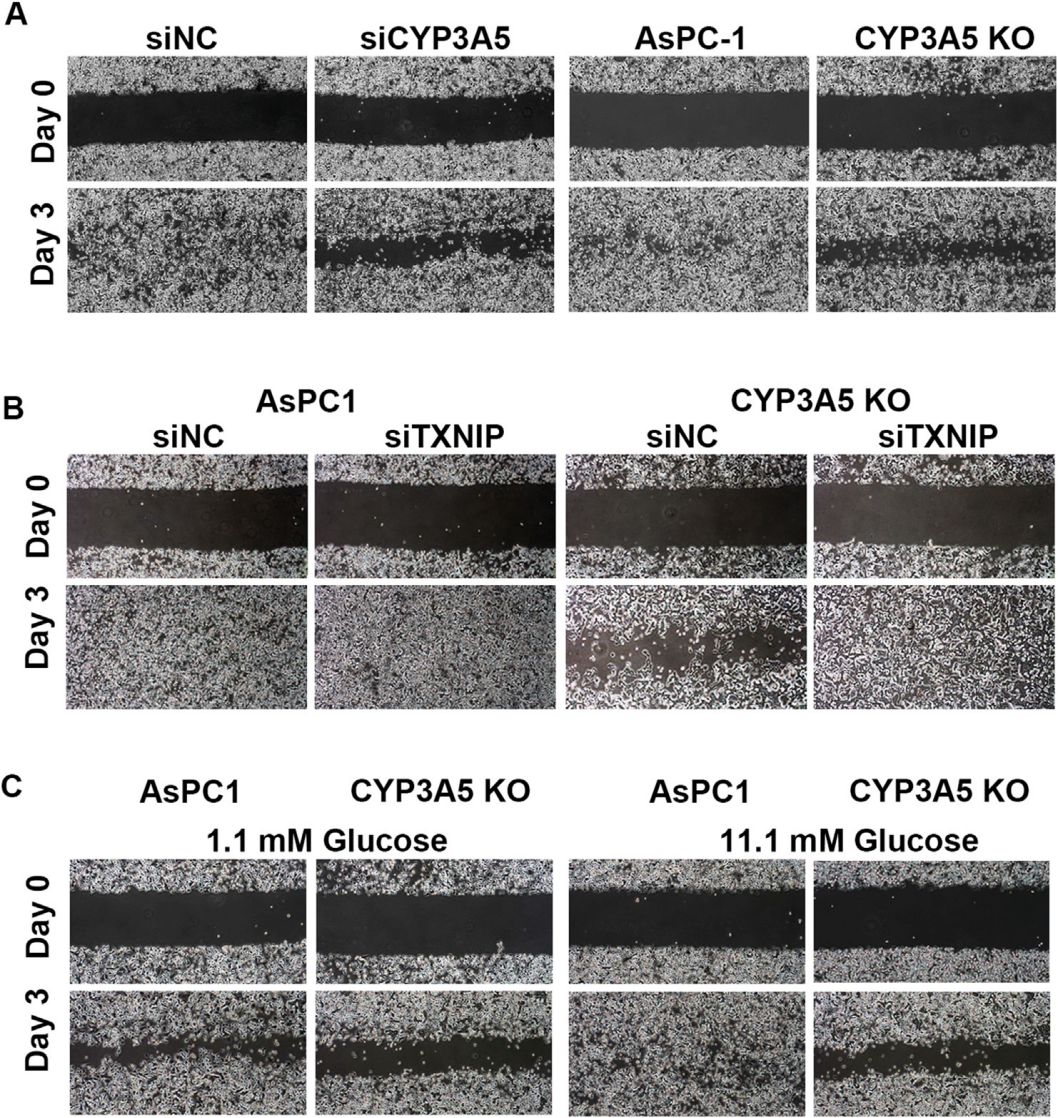
CYP3A5 down-regulation caused decreased cell migration in a TXNIP-dependent and glucose-dependent manner. **(A)** Results of wound-healing assay of AsPC-1 and CYP3A5-KD/KO cells. **(B)** Results of wound-healing assay of AsPC-1 and CYP3A5-KO cells after siTXNIP treatment. **(C)** Results of wound-healing assay of AsPC-1 and CYP3A5-KD/KO cells grown in medium containing 1.1 mM or 11.1 mM glucose.

Another significant phenotype we identified was cell migration. We used a cell wound-healing assay to assess the ability of cells to migrate. The results showed that after CYP3A5 knockdown/knockout, the rate of scratch closure was decreased (Fig. 7A), suggesting that CYP3A5 helped maintain normal migration of AsPC-1 cells. To confirm whether this was related to glucose metabolism, we knocked down TXNIP in AsPC-1 WT and CYP3A5-KO cells and found that, after the TXNIP protein levels were decreased, the ability of CYP3A5-KO cells to migrate was increased robustly (Fig. 7B). This result indicated that CYP3A5-regulated cell migration was TXNIP-dependent. To investigate further whether this effect was due to decreased glucose metabolism, we grew cells in a medium containing 1.1 mM or 11.1 mM glucose and found that the decreased glucose in the medium affected the migration of AsPC-1 cells but not that of CYP3A5-KO cells (Fig. 7C). In both conditions (1.1 mM and 11.1 mM glucose), the migration ability of AsPC-1 cells was stronger than that of CYP3A5-KO cells. These results suggested that the contribution of CYP3A5 to AsPC-1 cell migration was both TXNIP-dependent and glucose-dependent.

### CYP3A5 regulated glucose consumption via AKT–4EBP1–TXNIP signaling in AsPC-1 organoids-like culture

**Figure 8:**
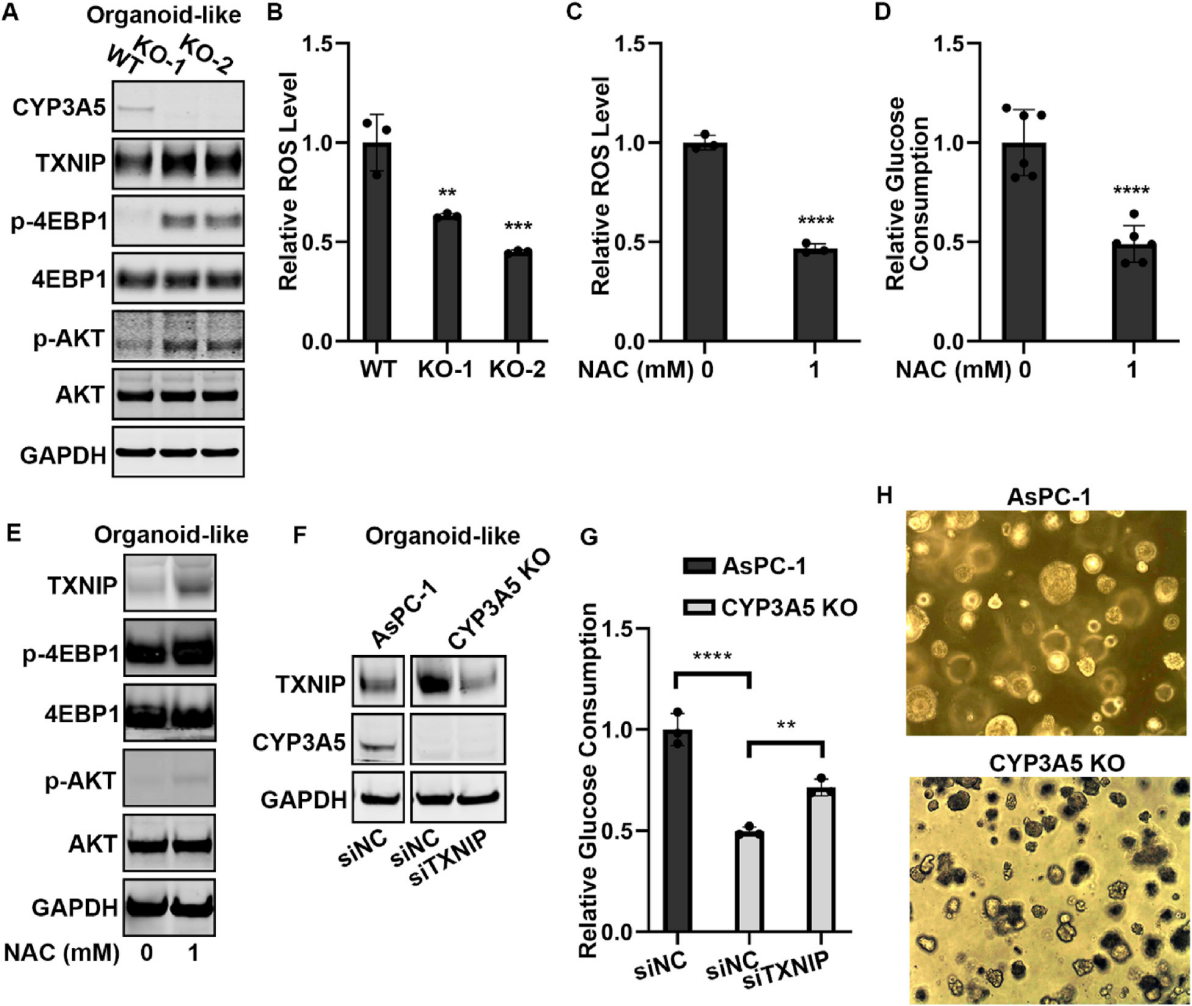
CYP3A5 regulated glucose consumption in AsPC-1 organoids via AKT–4EBP1–TXNIP signaling. **(A)** Western blot of CYP3A5, TXNIP, p-4EBP1, and p-AKT in AsPC-1 WT and CYP3A5-KO organoids. **(B)** ROS levels in AsPC-1 WT and CYP3A5-KO organoids. **(C)** ROS levels in AsPC-1 organoids after NAC treatment. **(D)** Glucose consumption in AsPC-1 organoids after NAC treatment. **(E)** Western blot of CYP3A5, TXNIP, p-4EBP1, and p-AKT in AsPC-1 organoids after NAC treatment. **(F)** Western blot of CYP3A5 and TXNIP in AsPC-1 WT and CYP3A5-KO organoids after siTXNIP treatment. **(G)** Glucose consumption in AsPC-1 WT and CYP3A5-KO organoids after siTXNIP treatment. **(H)** Images of AsPC-1 WT and CYP3A5-KO organoid-like cultures. Images were from culture on day 10.

2D cell cultures are a limited system with which to study glucose metabolism. We, therefore, investigated whether the mechanism that we had identified in 2D culture was also applied in a model that more closely resembled *in vivo* conditions. We grew AsPC-1 cells under organoid culture conditions to form organoid-like structures and found that, after CYP3A5 knockout, the TNXIP, p-4EBP1, and p-AKT protein levels were all up-regulated (Fig. 8A). We then examined whether CYP3A5-generated ROS were responsible for AKT–4EBP1–TXNIP signaling pathway regulation. We found that CYP3A5-KO organoid-like culture contained fewer ROS (Fig. 8B). After AsPC-1 WT organoid-like cultures were treated with NAC to decrease ROS (Fig. 8C), the glucose consumption was also decreased (Fig. 8D), whereas the protein levels of TXNIP, p-4EBP1, and p-AKT were all increased (Fig. 8E). To obtain further confirmation that AKT–4EBP1–TXNIP signaling was responsible for glucose metabolism regulation by CYP3A5 in organoid-like cultures, we knocked down TXNIP in AsPC-1 CYP3A5-KO organoid-like cultures (Fig. 8F). TXNIP down-regulation successfully rescued the decrease in glucose consumption caused by CYP3A5 knockout (Fig. 8G). Lastly, we examined if CYP3A5 knockout affected the growth of the organoid-like culture. Our data showed that CYP3A5 deletion decreased the size of the AsPC-1 organoid-like culture compared with the control group (Fig. 8H). This result further confirmed that CYP3A5 was an important factor for pancreatic cancer. In summary, these results indicated that in AsPC-1 organoids-like culture, CYP3A5 produced ROS, which inhibited the AKT–4EBP1–TXNIP signaling pathway. Decreased TXNIP was beneficial for increasing glucose uptake and organoid growth.

## Discussion

CYP3A5 is a member of the cytochrome P450 superfamily of enzymes that is involved in the metabolism of drugs, exogenous carcinogens, and endogenous molecules such as steroids.^5^, ^6^, ^7^, ^8^ Previous studies of CYP3A5 focused mainly on two aspects, namely the potential relation between CYP3A5 polymorphism and cancer risk or drug metabolism.^7^ The direct physiological role of CYP3A5 in pancreatic tumorigenesis remains unclear. In our current study, CYP3A5 expression was frequently up-regulated in tumor tissues and was associated with PDAC metabolism. We identified a novel role of CYP3A5 in PDAC glucose metabolism. CYP3A5 is highly expressed in PDAC cells and helps maintain the production of ROS, which inhibits AKT–4EBP1–TXNIP signaling. Decreased TXNIP contributes to enriching GLUT1 in the plasma membrane, resulting in higher glucose uptake to support the growth of PDAC cells. To our knowledge, this is the first study to evaluate how CYP3A5 regulates PDAC glucose metabolism.

The integrative metabolome and transcriptome profiling revealed a significant decrease in serine/glycine metabolism and the folate cycle after CYP3A5 knockdown. Serine is essential for the biosynthesis of proteins and other metabolites that sustain cellular proliferation, including nucleotides.^26^^,^^41^^,^^42^ Folic acid metabolism supports a broader set of transformations known as one-carbon metabolism, a generic metabolic process that serves to activate and transfer one-carbon biosynthetic processes, including purine and thymine synthesis and homocysteine remethylation.^60^^,^^61^ SHMT2 catalyzes the cleavage of serine to glycine and yields 5,10-methylene tetrahydrofolate, an essential intermediate for purine biosynthesis.^40^ In a 5,10-methylene-THF–dependent reaction, thymine synthase (TYMS) converts deoxyuridine monophosphoric acid (dUMP) to deoxythymidine monophosphoric acid (dTMP). After cells were treated with siCYP3A5, the SHMT2 protein levels were clearly decreased. This may explain why a cell cycle arrest happens after CYP3A5 knockdown. Besides the folate cycle, our RNA-Seq data also showed that steroid hormone biosynthesis, linoleic acid metabolism, and arachidonic acid metabolism changed after CYP3A5 knockdown. Considering that CYP3A5 takes part in lipid metabolism, it is worth investigating the relationship between CYP3A5 and lipid metabolism in cancer progression in the future.

Regulating glucose metabolism is one of the functions of TXNIP.^43^^,^^50^^,^^51^ TXNIP regulates both the transcription and trafficking of GLUT1, the latter by binding to plasma membrane GLUT1 and promoting its internalization in clathrin-coated pits. In PDAC cells, CYP3A5 is related to TXNIP protein synthesis. There is much more TXNIP protein in CYP3A5-deletion cell lines than in wild-type AsPC-1 cells. TXNIP is also involved in glucose uptake regulation in AsPC-1 cells and contributes significantly to decreasing GLUT1 in the plasma membrane. Knocking down TXNIP in CYP3A5-deletion cells can increase membrane-localized GLUT1 and rescues glucose uptake.

The mammalian CYP family contains heme-thiolate enzymes involved in the oxidative metabolism of various endogenous and exogenous lipophilic compounds.^7^ Poor catalytic cycle coupling of CYP results in continuous production of ROS,^58^^,^^62^ affecting signaling pathways and other cellular functions. Under certain conditions, such as when there is an invasion of exogenous substances, the exogenous metabolic process of CYP exposure to toxic compounds may be another important source of ROS. Originally characterized by their harmful effects on cells and invading microorganisms, ROS have been found to be involved in an increasing number of cell fate–determination and signal-transducing pathways.^63^, ^64^, ^65^ However, whether CYP3A5 contributes to ROS generation in PDAC cells was unclear. We have shown that CYP3A5 down-regulation indeed caused a decrease in the ROS level in AsPC-1 cells. ROS were also proved to be an upstream regulator of AKT–4EBP1–TXNIP signaling, which explains how CYP3A5 regulates TXNIP. We could also pharmacologically control ROS production, as compounds such as NAC can be applied to decrease ROS in our PDAC models, and the TXNIP protein level can be regulated in a dose- and time-dependent manner. The glucose uptake is decreased in NAC-treated wild-type cells upon ROS reduction.

## Conclusions

In summary, our results have identified a novel role for CYP3A5, a drug-metabolizing enzyme, in regulating the glucose metabolism of PDAC cells. High expression of CYP3A5 helps maintain the high level of ROS in cells, which inhibits AKT–4EBP1–TXNIP signaling. A decrease in TXNIP proteins contributes to increased GLUT1 in the plasma membrane, and higher glucose uptake is beneficial for the growth of PDAC cells, which is significantly decreased by CYP3A5 knockout. Collectively, these findings suggest a new strategy for treating PDAC.

## Author contributions

MS and TC conceived and organized the project. MS, HT, JW, HWL, ADH, J-HC, and JP designed the experiments and analyzed the data. QP, JY, and WCW analyzed data. MS and TC wrote the manuscript with inputs from all authors. All authors reviewed the final manuscript.

## Conflict of interests

Taosheng Chen is an Associate Editor for Genes & Diseases and was not involved in the editorial review or the decision to publish this article. All authors declare that there are no competing interests.

## Funding

Research reported in this publication was supported by the 10.13039/100000057National Institute of General Medical Sciences of the 10.13039/100000002National Institutes of Health under award number R35GM118041. The content is solely the responsibility of the authors and does not necessarily represent the official views of the National Institutes of Health.
