# Supplementary material for: CYP3A5 unexpectedly regulates glucose metabolism through the AKT–TXNIP–GLUT1 axis in pancreatic cancer
Source: Genes Dis. 2023 Sep 7;11(4):101079. doi: 10.1016/j.gendis.2023.101079 (PMC10980945; doi:10.1016/j.gendis.2023.101079)
Supplement: Multimedia component 2 [file mmc2.docx]

**Supplementary information:**

**Table S1** Metabolome profiling data (see uploaded Excel file).

**Table S2** qPCR primers and siRNA sequences.

| Gene |  | Sequence |
| --- | --- | --- |
| GLUT1 | pF | GGACAGGCTCAAAGAGGTTATG |
|  | pR | AGGAGGTGGGTGGAGTTAAT |
| TXNIP | pF | GTGATAGTGGAGGTGTGTGAAG |
|  | pR | CAGGTACTCCGAAGTCTGTTTG |
| PSAT1 | pF | ATCCGGGCCTCTCTGTATAA |
|  | pR | ATCCTGGTTAGGATGTGTTCATAG |
| PSPH | pF | GGCCTAGCGAAGATGAAGATAG |
|  | pR | CAGGTTCTTACGTCTCAACTCC |
| DHFR | pF | GCTAGCATGCAATGGTGTGA |
|  | pR | CCTGGTCCTAGCTACTTGGG |
| SHMT2 | pF | CTCTGAAGGGCACTCCTCAA |
|  | pR | GACATCGTCACCACCACTAC |
| MTHFD2 | pF | TGATCCTGGTTGGCGAGAAT |
|  | pR | ATGCTCTGGAAGAGGCAACT |
| MTHFD1L | pF | GCAGACTCCTGAAACAGACTAC |
|  | pR | CATGTCTCCAGCATTGCATAAC |
| SHMT1 | pF | CCAGTATGGTGGGAGGGTTT |
|  | pR | TCTCAGGTGGAGCAGGAAAG |
| TYMS | pF | CGCTACAGCCTGAGAGATGA |
|  | pR | ACTCCCTTGGAAGACAGCTC |
|  |  |  |
| siCYP3A5-5UTR | sense | 5' C.A.G.G.G.A.A.G.C.U.C.C.A.G.G.C.A.A.A.U.U 3' |
|  | antisense | 5' 5'-P.U.U.U.G.C.C.U.G.G.A.G.C.U.U.C.C.C.U.G.U.U 3' |
| siCYP3A5-3UTR | sense | 5' G.A.A.A.U.A.A.A.G.A.U.G.G.G.C.U.U.A.A.U.U 3' |
|  | antisense | 5' 5'-P.U.U.A.A.G.C.C.C.A.U.C.U.U.U.A.U.U.U.C.U.U 3' |


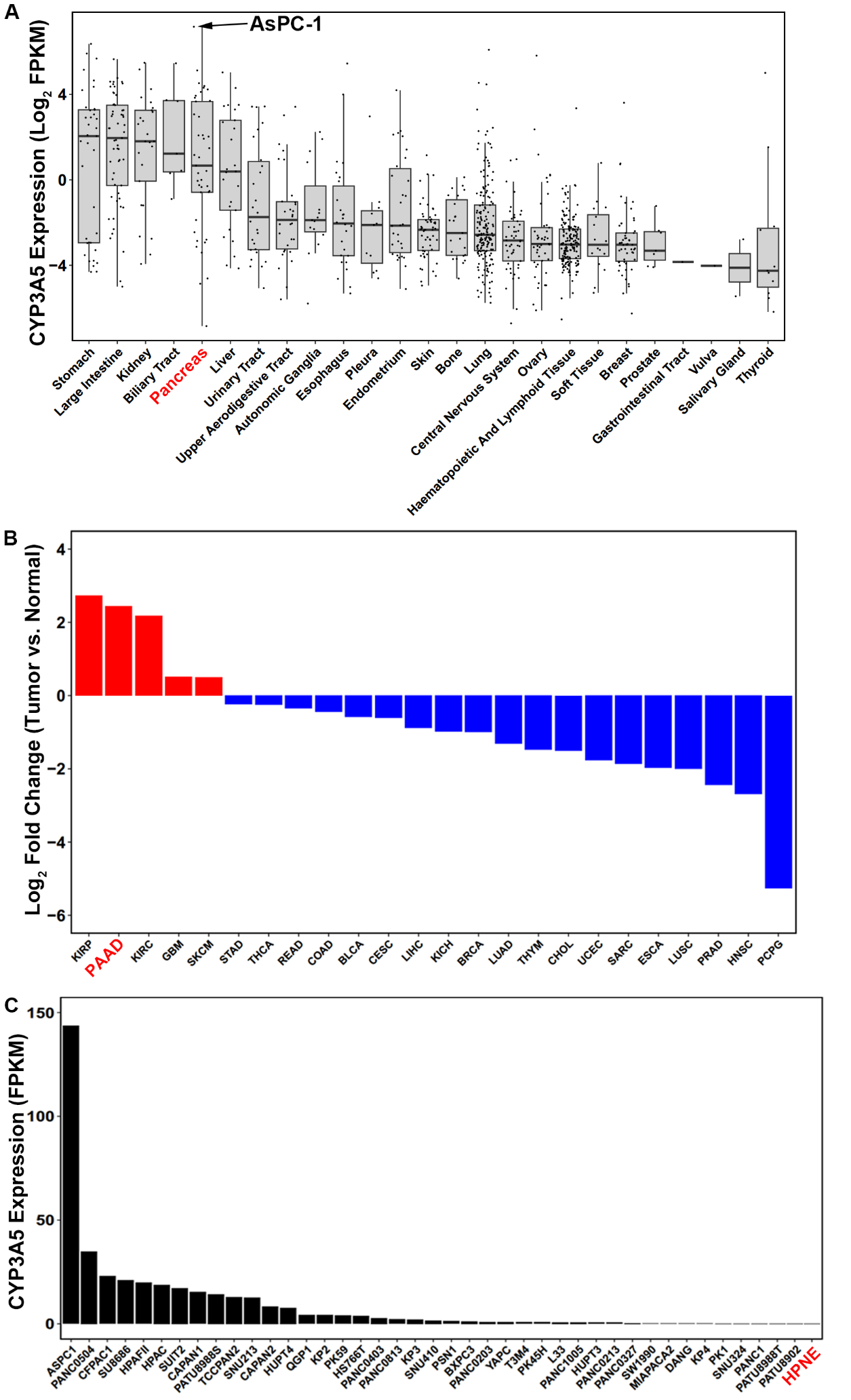


**Figure S1** CYP3A5 expression profiles of primary cancer types and pancreatic cell lines. **(A)** *CYP3A5* mRNA expression levels in different tumors based on Cancer Cell Line Encyclopedia data. AsPC-1 cell line has the highest CYP3A5 expression level. **(B)** Bar plot showing the fold change in *CYP3A5* expression between tumor and matched normal samples for 24 primary cancer types. The red bars indicate the cancer types in which the expression of CYP3A5 is higher in the tumor than in normal tissue, whereas the blue bars indicate those in which the expression of CYP3A5 is lower in the tumor than in normal tissue. **(C)** *CYP3A5* expression in 41 pancreatic cancer cell lines and 1 normal pancreatic ductal epithelial cell line HPNE (in red).


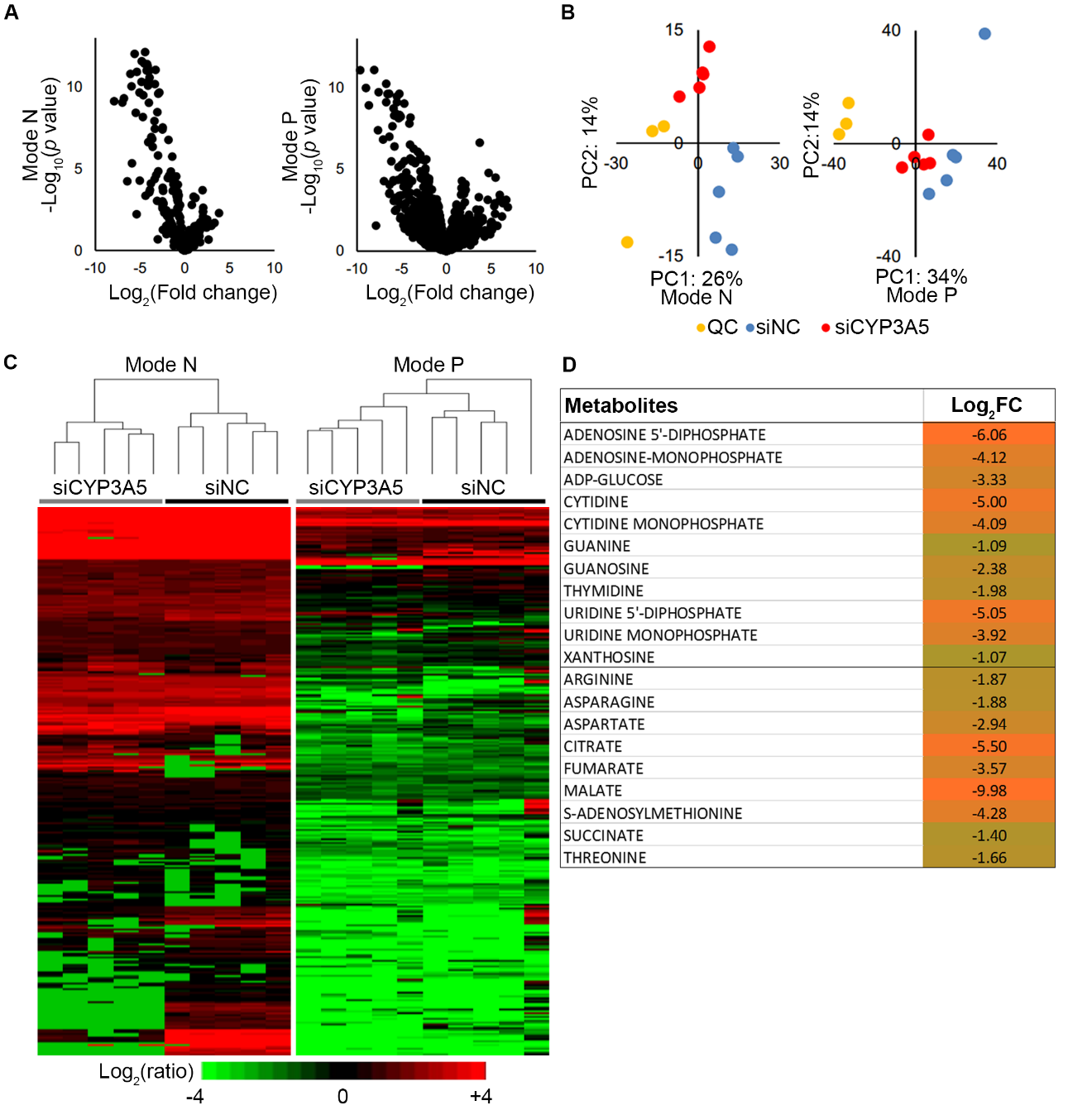


**Figure S2** Metabolome profiling of CYP3A5-knockdown AsPC-1 cells. **(A)** Volcano plot indicating metabolites that change significantly in CYP3A5-knockdown cells. **(B)** Principal component analysis of all identified and quantified metabolites. **(C)** Cluster analysis of all identified and quantified metabolites. **(D)** List of metabolites that change significantly (FC > 2, *P* < 0.05) after CYP3A5 knockdown in AsPC-1 cells.


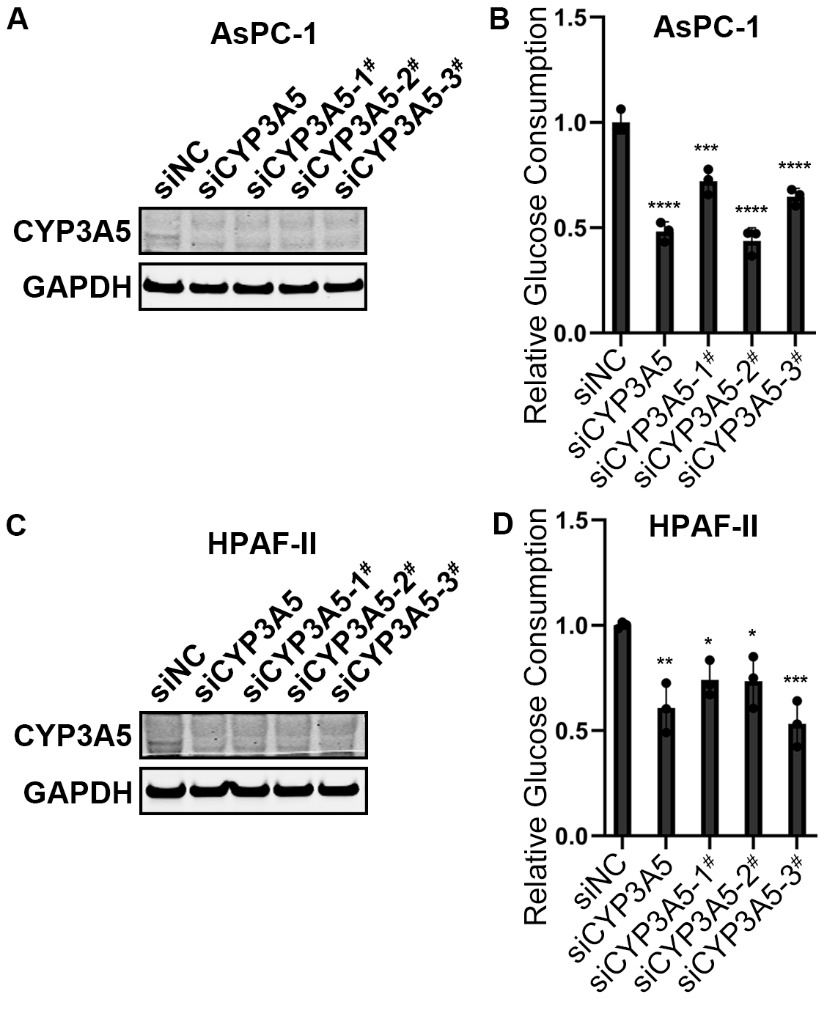


**Figure S3** CYP3A5 knockdown using individual siCYP3A5 siRNAs in AsPC-1 and HPAF-II cells. **(A)** Immunoblots showing levels of CYP3A5 in AsPC-1 cells after treatment with individual siCYP3A5 siRNAs. **(B)** Glucose consumption of AsPC-1 cells after treatment with individual siCYP3A5 siRNAs. **(C)** Immunoblots showing CYP3A5 levels in HPAF-II cells after treatment with individual siCYP3A5 siRNAs. **(D)** Glucose consumption of HPAF-II cells after treatment with individual siCYP3A5 siRNAs.


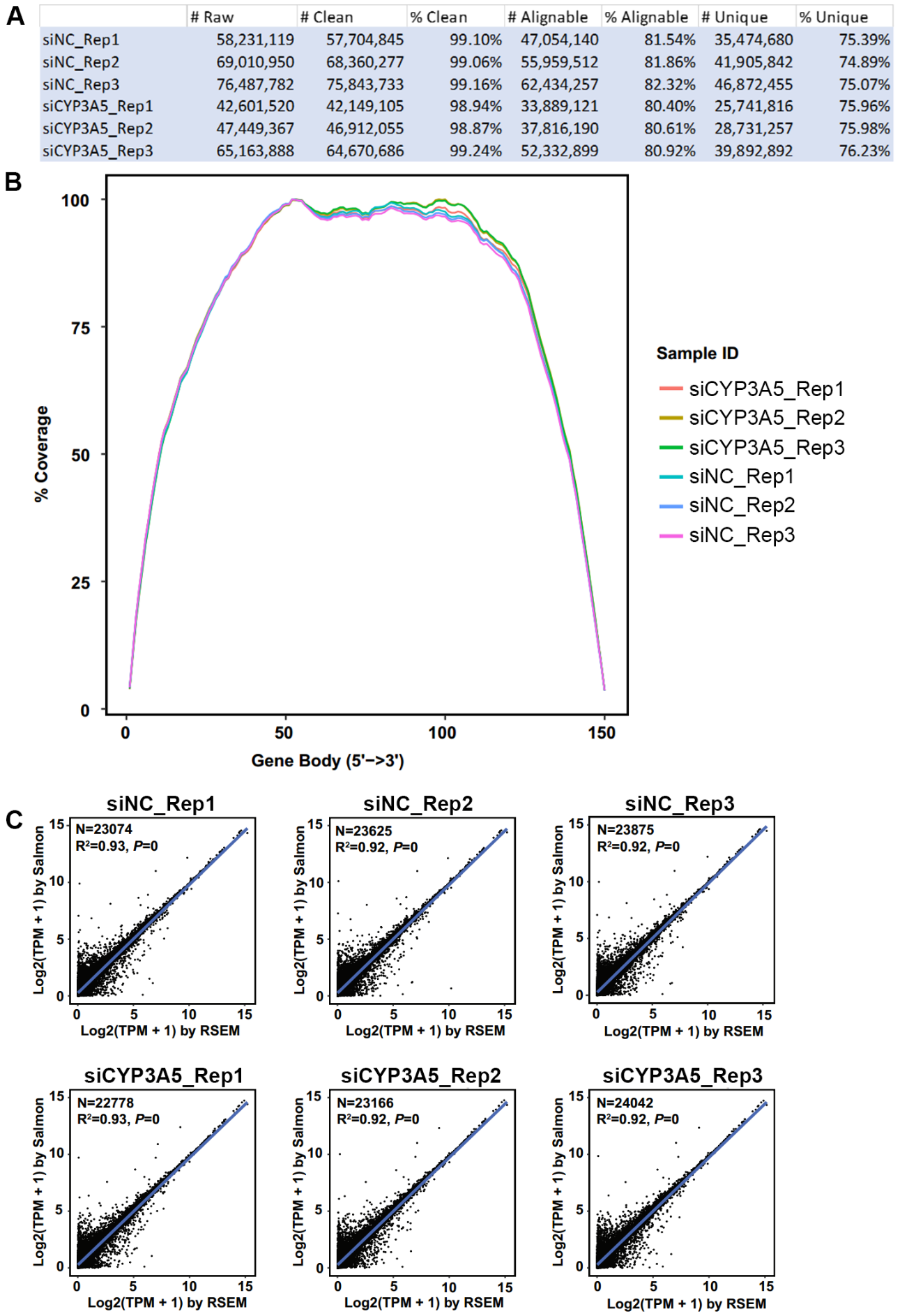


**Figure S4** Quality assessment of the transcriptomic analyses. **(A)** Alignment statistics of the RNA-seq data. **(B)** The gene body coverage statistics at a resolution of 150 bins per transcript­­. **(C)** The accuracy evaluation of gene expression quantification by RSEM. The Spearman correlation coefficients and *P* values were calculated from the genes co-identified by RSEM and Salmon, using the stats R package (v3.6.1).


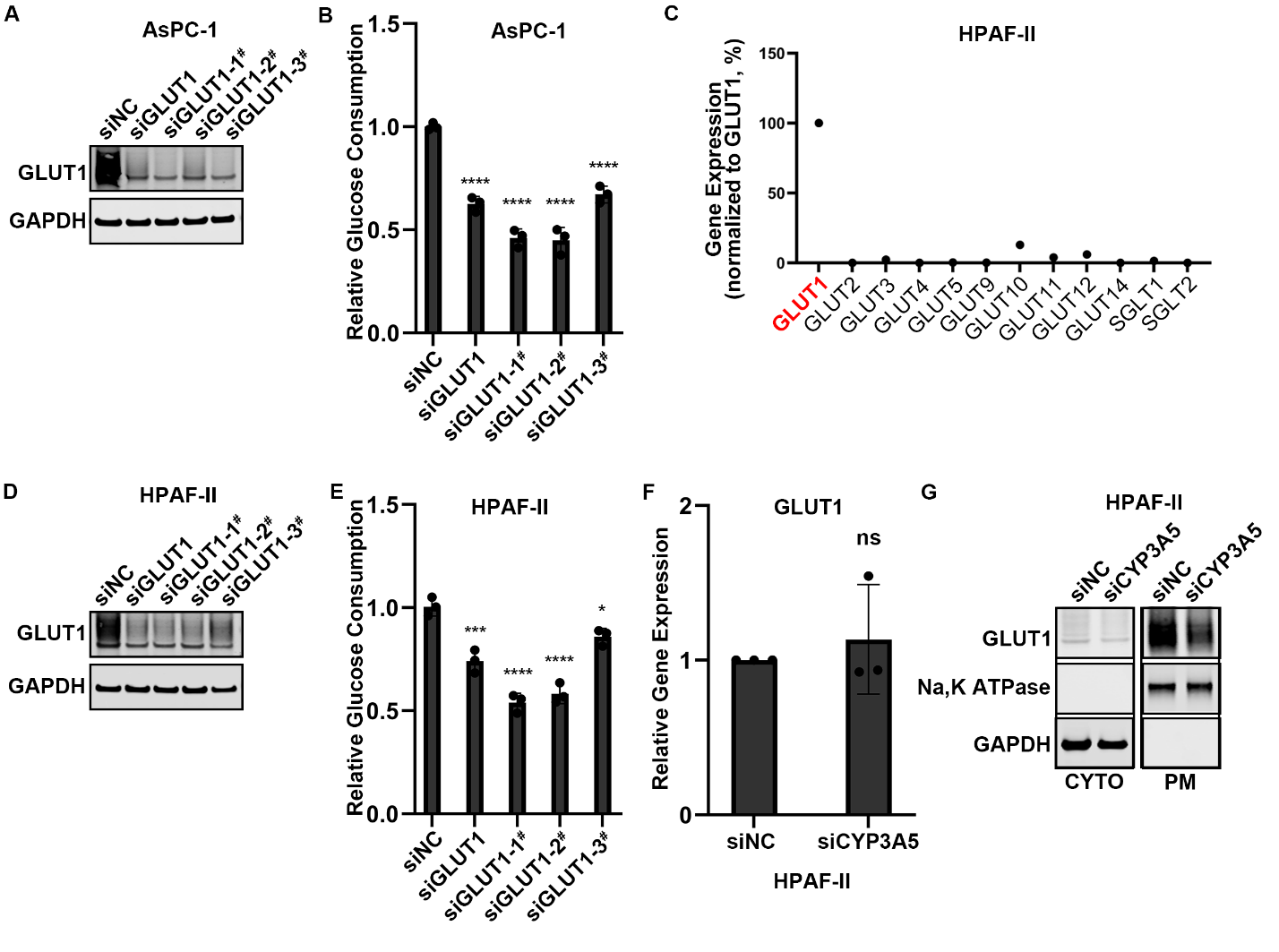


**Figure S5** Supplementary data for Figure 4. **(A)** Immunoblots showing the levels of GLUT1 in AsPC-1 cells after treatment with individual siGLUT1 siRNAs. **(B)** Glucose consumption of AsPC-1 cells after treatment with individual siGLUT1 siRNAs. **(C)** The expression level of various glucose transporters in HPAF-II cells based on RNA-seq data (GSE148998). **(D)** Immunoblots showing the levels of GLUT1 in HPAF-II cells after treatment with individual siGLUT1 siRNAs. **(E)** Glucose consumption of HPAF-II cells after treatment with individual siGLUT1 siRNAs. **(F)** qPCR analysis of GLUT1 mRNA levels in AsPC-1 cells after CYP3A5 downregulation. **(G)** Immunoblots showing levels of cytosol-localized and plasma membrane-localized GLUT1 in HPAF-II cells after CYP3A5 down-regulation.


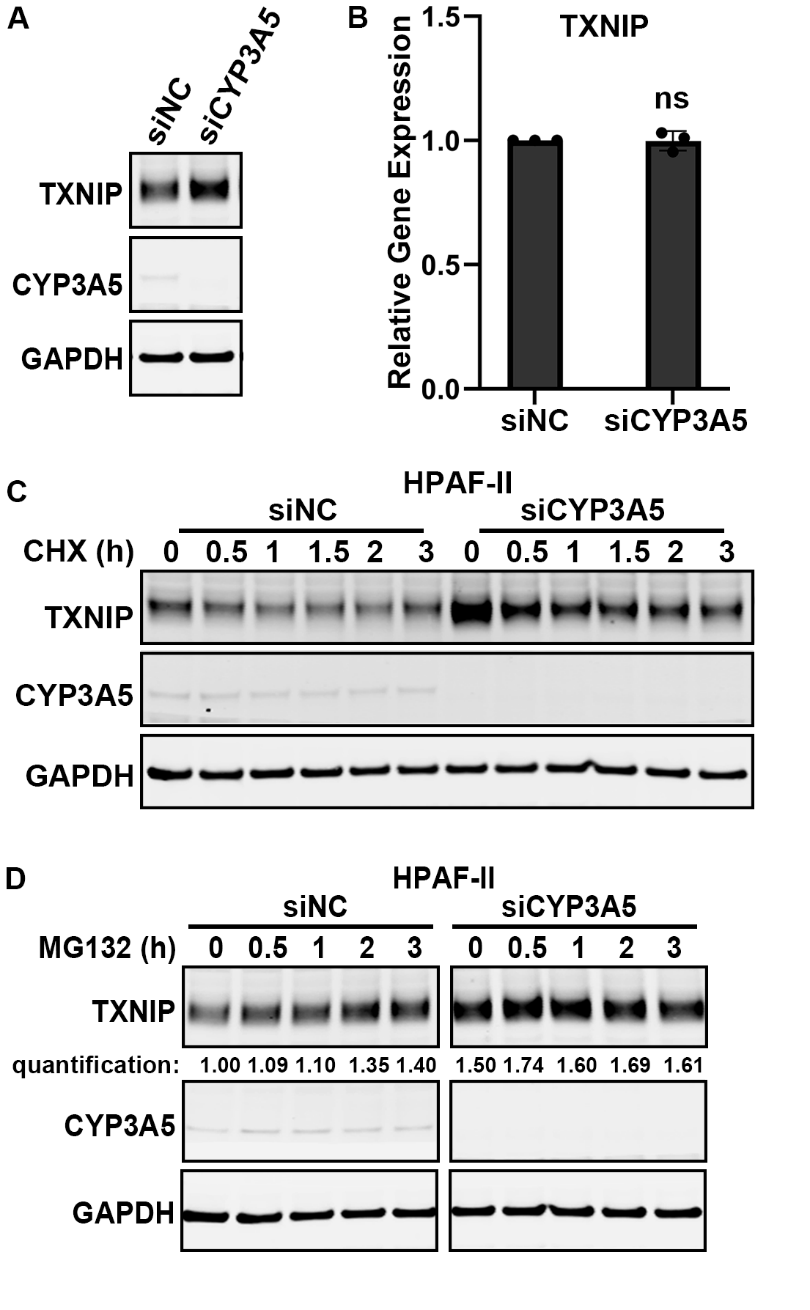


**Figure S6** CYP3A5 knockdown increased TXNIP translation in HPAF-II cells. **(A)** Immunoblots showing the levels of TXNIP in HPAF-II cells after CYP3A5 knockdown. **(B)** qPCR analysis of *TXNIP* mRNA levels in HPAF-II cells after CYP3A5 knockdown. **(C)** Immunoblots showing the levels of TXNIP in control and CYP3A5-KD HPAF-II cells after treatment with CHX (10 µM). **(D)** Immunoblots showing the levels of TXNIP in control and CYP3A5-KD HPAF-II cells after treatment with MG132 (10 µM).


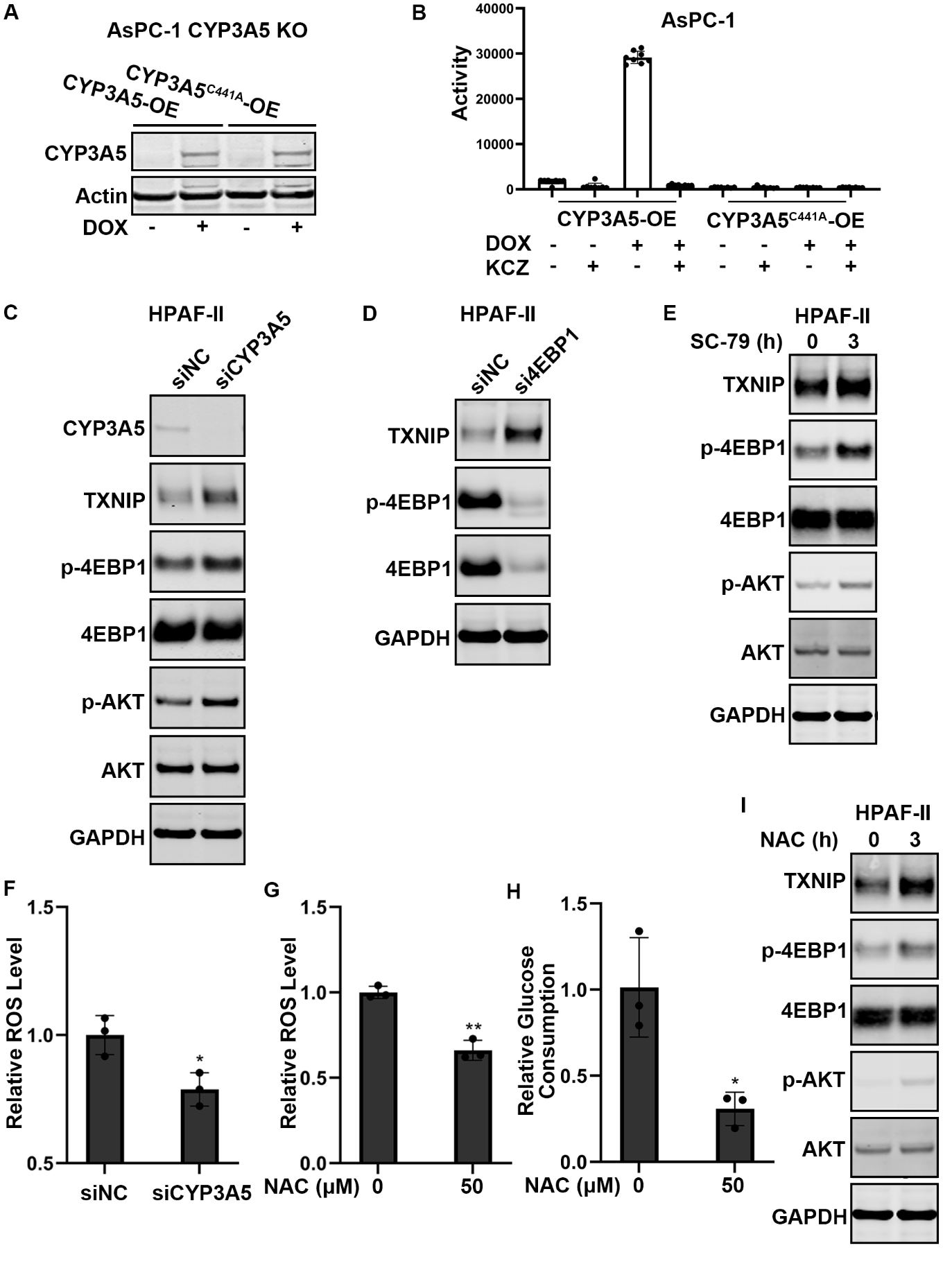


**Figure S7** CYP3A5-generated ROS are responsible for AKT–4EBP1–TXNIP signaling in HPAF-II cells. **(A)** Immunoblots showing the levels of CYP3A5 and CYP3A5^C441A^ proteins in CYP3A5-KO cells after treatment with DOX for inducing overexpression. **(B)** Enzymatic activity of CYP3A5 and CYP3A5^C441A^ proteins in CYP3A5-KO cells with CYP3A5/CYP3A5^C441A^ overexpression after treatment with DOX or ketoconazole. **(C)** Immunoblots showing the levels of CYP3A5, TXNIP, p-4EBP1, and p-AKT in HPAF-II cells after CYP3A5 knockdown. **(D)** Immunoblots showing the levels of TXNIP, p-4EBP1, and 4EBP1 in HPAF-II cells after si4EBP1 treatment. **(E)** Immunoblots showing the levels of TXNIP, p-4EBP1, and p-AKT in HPAF-II cells after treatment with SC-79 (10 µM). **(F)** The change in the ROS level in HPAF-II cells after siCYP3A5 treatment. **(G)** The change in the ROS level in HPAF-II cells after treatment with NAC for 3 h. **(H)** The change in the glucose consumption of HPAF-II cells after treatment with NAC for 3 h. **(I)** Immunoblots showing the levels of TXNIP, p-4EBP1, and p-AKT in HPAF-II cells after treatment with NAC (50 µM).


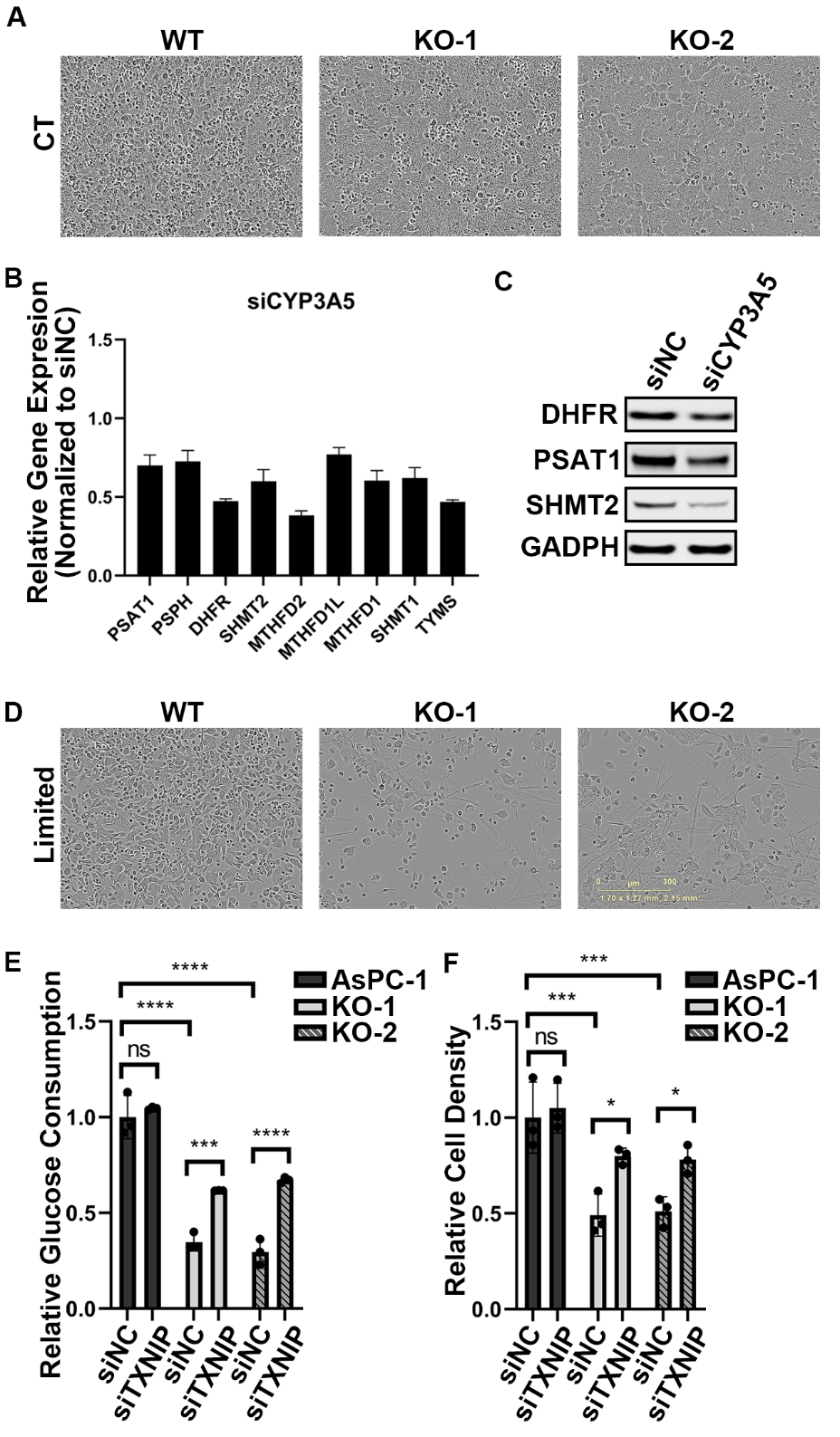


**Figure S8** CYP3A5 deficiency makes AsPC-1 cells more dependent on serine/glycine in the medium. **(A)** Images of AsPC-1 WT and CYP3A5-KO cells grown in normal medium (control medium, CT). **(B)** Results of qPCR analysis of genes related to serine/glycine and folate metabolism. **(C)** Immunoblots showing the levels of DHFR, PAST1, and SHMT2 in AsPC-1 cells after CYP3A5 knockdown. **(D)** Images of AsPC-1 WT and CYP3A5 KO cells grown in serine/glycine-limited medium (limited medium). Scale bar: 300 µm. **(E)** The change in the glucose consumption of AsPC-1 WT and CYP3A5-KO cells (grown in a limited medium) after siTXNIP treatment. **(F)** The change in the growth (based on cell density) of AsPC-1 WT and CYP3A5-KO cells (grown in limited medium) after siTXNIP treatment.
